# Patterns of treatment-seeking behaviors among caregivers of febrile young children: a Ugandan multiple case study

**DOI:** 10.1186/s12889-016-2813-7

**Published:** 2016-02-16

**Authors:** Rosemin Kassam, Richard Sekiwunga, Duncan MacLeod, Juliet Tembe, Eric Liow

**Affiliations:** School of Population and Public Health, Faculty of Medicine, University of British Columbia, 2206 East Mall, Vancouver, BC V6T 1Z3 Canada; Child Health and Development Centre, School of Medicine, Makerere University, Kampala, P.O.Box 7062, Uganda; The Islamic University, Mbale, Uganda

**Keywords:** Malaria, Treatment-seeking, Behavior, Child, Caregiver, Uganda, Case Study, Experiences

## Abstract

**Background:**

The vast majority of malaria deaths in Uganda occur in children five and under and in rural areas. This study’s exploratory case study approach captured unique situations to illustrate special attributes and aspects of treatment-seeking during a malaria episode.

**Methods:**

During August 2010, a qualitative exploratory study was conducted in seven of Butaleja District’s 12 sub-counties. Multiple case study methodology consisting of loosely-structured interviews were carried out with eight caregivers of children five and under in the local dialect. Caregivers were geographically distant and not known to each other. Interviews were translated into English and transcribed the same day. Data were analyzed using content analysis.

**Results:**

Of the eight cases, children recovered fully in three instances, survived but with deficits in three, and died in two. Common to all outcomes were (1) triggers to illness recognition, (2) similar treatment sequences and practices, (3) factors which influenced caregivers’ treatment-seeking decisions, (4) challenges encountered while seeking care at public health facilities, (5) cost burdens associated with managing malaria, (6) life burdens resulting from negative outcomes from malaria, (7) variations in caregiver knowledge about artemisinin combination therapy, and (8) varying perspectives how malaria management could be improved.

**Conclusions:**

Despite the reality that caregivers in Butaleja District generally share similar practices, experiences and challenges, very few children ever receive treatment in accordance with the Uganda’s national guidelines. To bring national practice into conformance with policy, three advances must occur: (1) All key stakeholders (those affiliated with the formal health system - public facilities and licensed private outlets, unlicensed drug vendors, and caregivers of young children) must concur on the need and the means to improve malaria management, (2) all health providers (formal and unlicensed) need to be engaged in training and certification to improve timely access to affordable treatment irrespective of a region’s remoteness or low population density, and (3) future public health interventions need to improve caregivers’ capacity to take the necessary actions to best manage malaria in young children.

**Electronic supplementary material:**

The online version of this article (doi:10.1186/s12889-016-2813-7) contains supplementary material, which is available to authorized users.

## Background

In 2010, there were an estimated 216 million episodes of malaria worldwide and 655,000 deaths [1]. Approximately 81 % of these episodes and 91 % of deaths were reported in the African Region, and nearly 86 % of the deaths were in children under five. Globally, malaria has been reported as the third largest cause of death in 2010, after pneumonia and diarrhoea [2]. Among those who survive, many are left with persistent anemia, impaired brain function, or paralysis [3]. The highest burden is in children because of their lack of immunity to the parasite [4–6]. Reducing child mortality from treatable diseases has therefore become a global priority, with African leaders at a summit in Abuja, Nigeria in 2000 pledging to halve malaria mortality by 2010 and two of the United Nation’s Development Goals focusing on reducing this unacceptable high level of mortality [5, 7–9]. One proposed declaration and plan of action from the African summit was to ensure by 2005 at least 60 % of those suffering from malaria receive prompt access to affordable and appropriate treatment within 24 h of the onset of symptoms [8]. However, despite several large-scale efforts to disseminate effective case management, the Abuja target continues to be elusive, with over 39,000 children each year dying of malaria in Uganda [10]. A large proportion of caregivers continue to delay seeking appropriate care for their children, many children are treated presumptively, and many others receive ineffective antimalarials [7, 11–15].

Several key impediments have been proposed in the literature to explain the gap between policy and practice within rural settings. At the service level - a large number of Ugandans have limited access to publicly funded health facilities, regulated pharmacies and trained health professionals, thereby relying on the unregulated private drug delivery sector that is untrained and unlicensed [16]. The popularity of the unregulated sector is further heightened because of their easy access, they do not require patients to wait in long queues or travel long distances, and they offer a range of drugs that can be purchased in any amount and without the need for prescription [15, 17, 18]. However, given that a large percentage of drugs and/or drug dosages dispensed by such vendors are inappropriate, their prominence presents a major challenge [12, 19]. At the household level - there are inappropriate self-management practices and an over-reliance on less effective antimalarials [15]. Such practices also influence what drug vendors sell, who often succumb to selling what the customer demands [18, 20]. At the community level - there are limited resources to support informed decision-making and demand for appropriate case management by caregivers [15, 16, 19]. Lastly, at the government level - resources are unavailable to enforce existing drug vendor regulations, which is compounded by the predicament that such enforcement would create in compounding the suffering of millions who do not have access to regulated health facilities and trained health professionals [16, 21].

A comprehensive strategy to improve caregivers’ management of malaria for young children will require more than policy changes. It will require an understanding of steps taken by caregivers when treating their children’s malaria, an explication of factors that predispose, enable and reinforce desirable treatment-seeking behaviors, and an understanding of elements which need to be enhanced and supported. This in turn, will require information on caregivers’ current treatment seeking patterns and their experiences with the health system. Accordingly, this investigation’s case study approach captures unique situations to illustrate special attributes and aspects of treatment-seeking during a malaria episode, including both good practices and weaknesses in care-giving. The exploratory case studies also reveal unrecognized factors influencing caregiver practices, help explain factors already identified, and determine factors that can be used as leverage points for defining a health promotion program.

This study’s objectives were to examine treatment-seeking patterns and to describe experiences of eight caregivers while managing the fever episode (presumed to be malaria by the caregiver) in their child of five years and under. Specifically, this research explored (1) the sequence of treatment steps taken by caregivers, (2) treatment options available to them when visiting sources external to their home, (3) challenges they encountered when seeking treatment, (4) financial and social burdens associated with malaria, and (5) caregivers’ knowledge about what is the best antimalarial for young children. The study sought to generate a broad perspective on treatment-seeking behavior by caregivers who treated their children and whose child experienced one of three different outcomes: (1) the child was cured (positive outcome), (2) the child survived but experienced a permanent disability (negative outcome), or (3) the child died (negative outcome). This study focused on the child’s most recent febrile episode resulting in the outcome of interest.

## Methods

This qualitative exploratory study was conducted in Butaleja District, Uganda as part of a baseline assessment for a larger study to examine caregivers’ treatment-seeking behaviors for children of five years and under with presumed malaria. A multiple case study methodology was implemented over a period of two weeks in August 2010, to understand caregivers’ treatment-seeking practices -- whether anticipated or emergent -- and to determine factors influencing these practices. Ethics approval for the project had been previously obtained from the Uganda National Council for Science and Technology, the Office of Research Ethics Simon Fraser University and the University of British Columbia British Columbia’s Behavioral and Research Ethics Board.

### Setting

The study investigated caregivers in Butaleja District located in rural eastern Uganda approximately 38 km southwest of the nearest large city Mbale and 210 km northeast of the capital Kampala [22]. Butaleja’s administrative structure consists of 10 sub-counties (mostly rural) and two town councils (designated urban centres). Based on the 2002 national census, the population of Butaleja District for 2010 was estimated at 206,200 [23]. The predominant ethnic group is the Banyole tribe and the predominant spoken language is Lunyole [22]. The district normally experiences two major rainfall periods between May and October, although it commonly experiences unpredictable rainfall patterns that result in severe flooding creating swamps, submerging gardens, destroying roads and leaving many families homeless [24]. The district’s economy is chiefly subsistence farming, with almost four-fifths of the population deriving its livelihood from crop production [24]. Poverty is generally a society-wide phenomenon, although women are likely to be poorer than men because they lack independent sources of income and so have less access to resources [23].

Malaria was the highest ranked cause of morbidity in the District in the period 2007–2009, with about eight in every 10 persons experiencing malaria/fever symptoms [23]. The public health infrastructure in Uganda is stratified into four levels: hospital at the district level, Health Centre (HC) III at the sub-county level, Health Centre (HC) II at the parish level, and Health Centre (HC) I constituting of a network of volunteer community health workers (CHWs) scattered in villages across the district [25]. At the time of this study the District had one public hospital located in Busolwe town council. While it was national policy to make cost free ACT available from all levels of the health system (levels I to public hospitals), supplied as the Coartem® brand, at the time of the study no CHWs were provided with ACTs [26]. Other ACT brands could also be purchased from private outlets, with subsidized pricing offered only at licensed private outlets through the Affordable Medicines Facility – Malaria (AMFm) scheme [27]. The District however had no pharmacies and only a few licensed drug shops located mostly in town centres and market areas. The largest fraction of private vendors included unlicensed private vendors distributed across the district who did not have formal training in the management of malaria [28]. Table 1 summarizes public and private outlets available at the time of the study in the sub-counties and parishes where study participants resided.

**Table 1:** Distribution of public health and private outlets in sub-counties (parishes) where cases resided

Cases (CS): sub-county (parish)→ Outlet type ↓	CS 01: Himutu (Kaiti)	CS 02: Busaba (Buwihula)	CS 03: Nawanjofu (Bingo)	CS 04: Naweyo (Kaiti)	CS 05: Butaleja (Nakwasi)	CS 06: Naweyo (Kaiti)	CS 07: Naweyo (Kaiti)	CS 08: Busabi (Malangha)
Health centre III	--	--	1	--	1^a^	--	--	--
Health centre II	--	--	1	--	1	--	--	1^a^
Private clinic	--	--	--	--	--	--	--	--
Pharmacies	--	--	--	--	--	--	--	--
Drug shop (Licensed)	--	2	--	--	--	--	--	--
Drug shop (Unlicensed)	--	4^a^	3	5^b^	--	5^a^	5^b^	--
General shop (Unlicensed)	--	--	--	1	--	1	1	--
Market stall (Unlicensed)	--	--	--	--	--	--	--	--
Kiosk (Unlicensed)	--	--	--	--	--	--	--	--
Mobile vendor (Unlicensed)	--	--	1	--	--	--	--	--
Total outlets		6	6	6	2	6	6	1

^a^One drug shop located in the village where caregiver resided

^b^Three drug shops located in the village where caregiver resided

### Study sample

The target population consisted of caregivers who resided within Butaleja District, had at least one child five years and under, who provided most of the children’s day-to-day care, were able to understand and speak the local dialect of Lunyole, and who were willing and able to provide consent to participate in the study. Using purposive sampling, caregivers were recruited who had unique experiences managing malaria in a child of five years and under, resided in different regions of the District, and who were willing to discuss their experiences. For all eight children, malaria as the cause for the fever episode was established by their caregivers and/or health providers based on presumptive diagnosis. As part of an earlier household survey process, the research team had identified households with caregivers who appeared eager to share their treatment-seeking experiences along with an annotation of whether their child had recovered uneventfully from a febrile episode or had experienced a negative outcome. At the time of the survey, these caregivers had also been asked if: (1) they would agree to be contacted again for a more in-depth discussion about their experiences with managing malaria in one of their youngest children, and (2) for their permission to share their name and contact information with the study’s recruitment team. A list of potential caregivers for the case study was then created with caregivers classified according to their experiences: (1) those who sought treatment external to their home and the child improved or those who did not seek external care and the child survived, (2) those who sought treatment external to their home but the febrile illness persisted resulting in some form of disability, (3) those who sought treatment external to their home but the treatment failed and the child died. Names of potential caregivers from each of the categories were written on slips of paper, folded, put it in a container and drawn randomly using a ballot process. Caregivers were subsequently contacted by the study team and invited to participate. The first eight consenting caregivers became the case study participants.

### Data collection

Case study interviews were carried out by two trained research assistants recruited from the District and fluent in verbal and written English as well as Lunyole. Each research assistant was responsible for conducting and recording the interview. The study team traveled to the individual caregivers’ homes to conduct the interviews. While an interview guide was provided to the research assistants for areas to explore, the interviews were largely unstructured and pursued information provided by caregivers rather than adhering to a rigid protocol. Research assistants offered occasional prompts to keep the conversations flowing about the child’s history preceding the fever episode, signs and symptoms during the fever episode, treatment they sought – where and why, challenges they faced, length of the episode, child’s status following the episode, possible improvements to caregiving; nevertheless, each case study was a conversation rather than a structured interview. Data from case studies were collected in the local language over two to four visits, each lasting no more than one hour. Because Lunyole is not a written language, conversation was verbatim translated into English by the research assistants as they took interview notes. To ensure quality and accuracy, all conversations were audiotaped with the permission of all participants. Components of the conversation dealing with medications used during the child’s treatment were verified by asking caregivers to identify photos of each drug or medicine from a poster-sized collection of images of known drugs and their various brand names thus minimizing problems of recall or unknown drug names. At the end of each day, the research assistants met with a senior member of the research team to debrief and share critical perspectives, and all transcriptions were reviewed by the team within 24 h of completion.

### Analysis

Qualitative data analysis techniques were used to analyze the data. All caregivers’ responses were transcribed onto Microsoft Word documents, formatted, and entered into a qualitative data analysis computer program (QDA Miner) for subsequent coding, thematic analysis, and interpretation. All transcripts were reviewed and coded using a constant comparative method. As a first step, three of the authors independently reviewed all transcripts line-by-line identifying both anticipated and emergent themes. The themes were then discussed, debated, and where necessary relabeled or regrouped into new themes using open, axial, and select coding techniques [29]. Once the labeling was completed, the themes were grouped into categories and analyzed consistent with the study’s overall objectives for specific indicators, patterns, and trends in both anticipated and emergent outcomes. Qualitative research issues of trustworthiness and replicability (validity and reliability) were verified by examining the number of times each theme was mentioned by different caregivers – either in the affirmative or negative. Furthermore, issues of trustworthiness and confirmability were assured by using multiple investigators in the analysis process and with repeated cycles of theme identification, definition, and reconciliation until all research team members concurred on the coding system and ultimately agreed on which utterances represented which codes [30]. Subsequently, to enable comparison across cases, select data was rendered into tables to illustrate the sequence of treatment steps taken by caregivers, their experiences with external sources, and their knowledge about antimalarial medicines.

## Results

Eight caregivers (seven female and one male) participated in the case studies (CS). Children generally experienced one of three outcomes from their febrile illness and treatment received: (1) a positive outcome where the child improved (CS 06, 07, 08), (2) a negative outcome where the child survived but experienced an irreversible disability (CS: 01, 02, 03), or (3) a negative outcome where the child died (CS: 04, 05). Caregivers’ and their index children’s demographic characteristics are summarized in Table 2. Caregivers’ ages ranged from 22 to 45 years, and their index children’s ages ranged from two months to four years at the time of the febrile episode. All caregivers were peasant farmers living in monogamous households. Two of the caregivers had completed some level of post-secondary education, five had some level of primary schooling, and one had no formal education. Five of the caregivers reported being protestant, one was Muslim, and two made no mention of their religious affiliations. The eight caregivers represented seven of the 10 sub-counties in Butaleja District.

**Table 2:** Caregivers’ and index child’s demographic characteristics

Cases → Demographics ↓	CS 01	CS 02	CS 03	CS 04	CS 05	CS 06	CS 07	CS 08
Household Information								
Sub-county	Himutu	Busaba	Nawanjofu	Naweyo	Butaleja	Naweyo	Naweyo	Busabi
Parish	Kaiti	Buwihula	Bingo	Kaiti	Nakwasi	Kaiti	Kaiti	Malangha
Caregivers’ Demographic Characteristics								
Age	30 years	28 years	42 years	43 years	22 years	30 years	45 years	25 years
Education	Partial primary	Partial secondary	Partial primary	Partial primary	Partial primary	None	Partial secondary	Partial primary
Occupation	Farmer	Farmer	Farmer	Farmer	Farmer	Farmer	Farmer	Farmer
Religion	Protestant	Protestant	Protestant	Not Available	Not Available	Muslim	Protestant	Protestant
Marriage status	Yes Monogamy	Yes Monogamy	Yes Monogamy	Yes Monogamy	Yes Monogamy	Yes Polygamy	Yes Monogamy	Yes Monogamy
Spouse’s occupation	Farmer	Mechanic	Farmer	Farmer	Farmer	Farmer	Farmer	Teacher
No. Children	5	2	9	7	4	6	6	5
Index Child’s Demographic Information								
Gender	Male	Female	Female	Male	Female	Male	Male	Male
Age	3 years	4 years	3 years	1 year	2 years	2 months	1 year	1 year

### Case study summaries (Additional files: Table S1 and Table S2)

#### Case study (CS) 01

Date of episode not discussed). At 10 pm, the caregiver noticed that her child was feverish and convulsing. She initiated home management with Coartem®, Panadol® and physical supportive therapy using a wet cloth to decrease the fever before taking the child to a public health facility the following morning. The public facility (Kangalaba HC III) offered no treatment but instead referred the child to a hospital in the neighboring district of Mbale for blood transfusion. At the hospital, the child was admitted and given six quinine injections over three days and two-week worth of quinine syrup. Post-discharge the child’s vision worsened and the left hand became lame. Over the next month, the caregiver took the child to visit optometrists and doctors in the district of Tororo and was told the child had received too much quinine and nothing could be done to help the child.

#### Case study (CS) 02

(Episode two years prior interview). At 4 am, the caregiver noticed her child was ill with fever and convulsing. The caregiver initiated home management with traditional remedy (smearing pounded onions of the child’s body and giving the child to drink some), and then at 10 am took the child to a public health facility (Busaba HC III). At the public facility the child was given a blood test, Septrin®, Panadol®, and quinine tablets, and was told to buy additional quinine syrup from a private drug vendor. Because the child continued to convulse, the caregiver continued to give quinine syrup daily for about a year, while periodically returning to the same public facility as well as visiting the local drug shop to find some relief for her child’s convulsions. However, no further tests or different treatments were offered. When the child suddenly stopped speaking she stopped the quinine and reverted back to using traditional remedies (bathing in mululusa and tomato leaves) for a period of one month. She eventually stopped using the traditional remedies when the child did not respond and, a year later, at the suggestion of a friend, she inquired about anticonvulsant tablets at a HC III and got referred to the main hospital where the child received additional quinine and convulsion tablets. The child now receives anticonvulsant tablets daily which the parents obtain from the hospital on a weekly basis. While the frequency of convulsions decreased, the child lost the ability to speak.

#### Case study (CS) 03

(Episode two months prior interview). At 5 pm, the caregiver noticed her child had a swollen stomach and was feverish, vomiting and convulsing. She initiated home management with tradition remedy (smearing pounded onions on the child’s body) to lower the child’s fever, and at 10 am the next day visited a private drug shop in Busolwe. The drug vendor assessed the child to have cerebral malaria and gave the child an injection and various tablets including Panadol®. The caregiver returned the next day for the same medicines. The child’s fever gradually improved after two-weeks, but to this day continues to experience convulsions and a swollen stomach.

#### Case study (CS) 04

(Episode five years prior interview). At 4 pm, the caregiver noticed her child was ill with fever and short of breath. She initiated home management with Panadol®, Septrin® and supportive therapy using a wet cloth. The following day at 7 am when she saw the child’s condition had worsened, she took the child to a public health facility (Nabiganda HC II). No treatment was given at the facility, instead the child was referred to a hospital in the neighboring district of Mbale for blood transfusion which took two and a half hours to reach. When the child arrived at Mbale the doctors ordered blood tests, but during the three hours while the tests were being run the child died.

#### Case study (CS) 05

(Episode five months prior interview). At 5 pm, the caregiver noticed her child was ill with fever, vomiting, coughing and experiencing diarrhea. She initiated home management with supportive therapy using a wet cloth to cool the child’s fever before visiting a public health facility at 6 pm (Nakwasi HC III). At the health facility the child received Coartem® and was told to buy quinine syrup from a drug shop. Despite treatment, the child continued to worsen. The caregiver returned to the HC twice over the subsequent two months without any improvement. Three months later, the caregiver took the child to a private drug vendor because the public facility was closed. There the child was given tablets to treat worms, the child appeared to improve after the initial dose but quickly turned for the worst and died.

#### Case study (CS) 06

(Episode two months prior interview). In the morning the caregiver noticed that her child was short of breath but decided to do nothing until the next morning when the child’s condition worsened, at which time she visited a private drug shop. The drug vendor gave the child Aspirin^©^ for three days which the caregiver administered. No additional medicines were purchased because the household could not afford to buy more medicines. The caregiver said it took two weeks for the fever to resolve on its own.

#### Case study (CS) 07

(Episode two months prior interview). At 9 am, when the caregiver noticed the child was ill with fever, chills, and vomiting, he initiated home management with Panadol®. Later the same day when there was no improvement the child was taken to a private drug shop. The drug vendor took a blood test, gave the child two quinine injections, and the child gradually improved over a few days eventually returning to normal.

#### Case study (CS) 08

(Episode two months prior interview). At 7 am, the caregiver noticed her child was feverish and not being her usual self. She initiated home management with Coartem®, Panadol® and mululusa, and used supportive therapy with wet cloth to reduce the fever, but the child’s conditioned continued to worsen. At 10 am the next day, the child was taken to a public health facility (Muhuyu HC II) where the child was given more Coartem®, Panadol®, some kind of injection and Rene Coldease®. The child’s illness was cured in 3–4 days.

### Case study findings

Eight broad themes summarize caregivers’ treatment seeking patterns for the reported febrile episodes, their experiences, what they knew and what they learned. These include: (1) how caregivers’ recognized their child’s illness, (2) the sequence of treatments sources and treatment practices involved, (3) factors which influenced caregivers’ treatment-seeking decisions, (4) challenges they encountered with seeking care at public health facilities, (5) the burden of cost associated with managing malaria, (6) the burden of living with a negative outcome from malaria, (7) caregivers’ knowledge about ACT, and (8) caregivers’ perspectives of how management of malaria could be improved. Quotes that best illustrate the above themes and represent views from all three outcomes of interest are presented. Additional file 1: Table S1 illustrates the sequence of care received by the child, the initial symptoms and the time of day when symptoms were first noticed, the different external sources visited and treatments received during these occurrences, and outcome(s) at the end of each treatment phase. Additional file 2: Table S2 outlines caregivers’ treatment-seeking experiences when seeking treatment from an external source for the episode of fever in question. Tables provide a “running description” of history, treatments, caregiver experiences, and subsequent reflection. Entries in the tables are arranged in order of the episode’s outcome: child recovered, child survived but with deficits, or the child died.

### Recognition of malaria

“Recognition” examines how caregivers identified their child was suffering from malaria. It captures the most common symptoms recognized, the time of day when symptoms were first noticed, and how this affected caregivers’ treatment–seeking decisions (Additional file 1: Table S1).

#### Symptoms and time of recognition

With the exception of one caregiver, “fever” or “hot body” was the most common symptom mentioned, followed by coughing/congestion observed in cases that had positive outcomes as well as ones where the child died (CS: 04, 05, 06, 08). Convulsion was mentioned only by those caregivers whose child had survived with a negative outcome (CS: 01, 02 and 03), and it commonly accompanied fever. Some of the other primary symptoms mentioned included “red eyes”, “diarrhea”, “swelling of the body”, “pain within body”, “the child not eating”, “vomiting”, and “making excessive noise”. The responses from the case studies showed that for all three children with positive outcomes (CS: 06, 07, 08), their symptoms were noticed early in the morning. Conversely, for those children who died or survived with a negative outcome, their caregiver first noticed their symptoms at night.

### Treatment actions

The steps taken by caregivers to treat their children once the initial symptoms were noticed were termed *Treatment Actions*. Included in this theme are the different steps taken by caregivers, treatment sources accessed, types of treatments received, and outcome at the end of each treatment occurrence (Additional file 1: Table S1). The multiple steps taken by caregivers represent treatment failures and attempts to seek additional care to resolve the febrile episode, as well as manage adverse outcomes from the illness. The treatment sources accessed were classified as either home management or external treatment which typically included a public health facility or a private drug vendor. Home management was defined as any care initiated from the home setting with resources within the household, such as physical supportive care, western medicines, and/or traditional remedies. Time to seeking external treatment or where treatment was sought appears to have had little influence on the outcome of the illness. Treatment at public health facilities was rarely successful, with four of the five children who visited a public health facility as part of their first external care experiencing a negative outcome. Of the two children who died, one died at a referral hospital and the other at home after several visits to a public health facility with repeat treatment failure.

#### First action

All but one caregiver (CS: 06) initiated treatment with some form of home management soon after noticing fever. The one caregiver (CS: 06) who did not initiate home management, opted to simply wait and observe the child for the first day, and initiated the first treatment on the next day with Junior Aspirin® obtained from a private drug vendor when the child’s symptoms worsened. Across the seven cases, three types of home management treatments were seen: western medicines, physical supportive therapy, and traditional remedies. Examples of physical supportive care included bathing the child in cool water, washing the child with a cool cloth or sponging the child to relieve fever. Western remedies used in home management included Coartem®, Panadol®, Septrin®, and quinine syrup. Traditional remedies included smearing onion paste over the ill child or sitting the child in a bath of herbs such as mululusa.

Western medicines were the most popular home management, with all positive outcome cases (CS: 07, 08) and half of the negative outcome cases (CS: 01, 02, 04) receiving some type of medicine. There is no strong pattern separating the cases with positive and negative outcomes in terms of which western medicines were given. Of the three children given an antimalarial, two received Coartem® (one with a positive one with a negative outcome) and one with a negative outcome received quinine. On the other hand, supportive care only was used mostly by those who experienced a negative outcome (CS: 01, 03, 04, 05), with only one positive outcome case reported to have used supportive care (CS: 08). Children were given supportive care with/out western medicines. Traditional remedies were only used by three caregivers (CS: 02, 03, 08) whose children later went on to experience a negative outcome, only one used it in combination with a western medicine (quinine).

Just over half of the caregivers representing children with a negative outcome (CS: 01, 02, 03, 05) reported initiating home management only as a “first aid” measure, with the intent to seek subsequent treatment from an external source first thing in the morning. Whereas the remaining caregivers (CS: 04, 07, 08) indicated they decided to seek additional care from an external source only after noticing their child’s condition had worsened.

*I had Panadol® and I gave her, and I still saw that her condition was still bad. I tried to take her to the health unit. (CS: 07)*

*I did not go to the health unit on that very day because I had trusted that bathing him with mululusa and giving him Coartem® and Panadol® would help. When I got to know that the fever had worsened, when it dawned to morning, I took him to the health unit. (CS: 08)*

Caregivers started treatment at home because they had western medicines at home and trusted that they would cure the child:

*I knew that this [Panadol®] and this [Septrin®] treats … fever.… In the health unit, the health providers teach us [to] use [Panadol® and Septrin®]. (CS: 04)*

*I know that Panadol® could reduce a bit the pain in the body…. The health provider told me, that this one [Panadol®] reduces painful body. (CS: 07)*

#### Second and subsequent actions

Additional file 1: Table S1 shows that all second and most subsequent actions took place externally and outside the home for all caregivers. Case 02 was the exception, as she reverted from visiting external sources and obtaining quinine treatment during subsequent actions to using traditional remedies from home when her child stopped speaking and she found western medicines were not helping. Three of the caregivers (CS: 02, 05, 07) reported visiting an external source within the same day, whereas the remaining caregivers went the next day. However, all caregivers whose child experienced a negative outcome reported seeking care within 24 h of noticing initial symptoms. The most common second action (and first external source) for five of the caregivers involved going to a public health facility, with four opting for the nearest facility (CS: 02, 04, 05, 08). Of the cases with a positive outcome, only one (CS: 08) was taken to a public health facility, the other two received treatment at private drug shops. On the other hand, all but one case with a negative outcome (CS: 01, 02, 04, 05) sought their first external treatment from a public health facility. All children who were subsequently referred to higher level health facility or those who required further treatment experienced a negative outcome. The most common medicines given or prescribed by external outlets were Panadol® tablets and/or some form of quinine. Only two children received Coartem® and both were at a public facility. Additional file 2: Table S2 illustrates caregivers’ experiences when seeking external care and whether or not they accessed their nearest public health facility.

#### Treatment in an ensuing malaria episode

While the intent of this study was to focus principally on the single febrile episode leading to the outcome in question, some of the illnesses never subsided and children either continued to experience mild fever or experienced frequent recurrent infections, which made it difficult to separate the original episode from new infections. As such, half of the cases (CS: 03, 06, 07, 08) mentioned their treatment-seeking experiences with a subsequent febrile episode as well. Two caregivers (CS: 03, 07) sought external care from a drug shop as they had done in the first episode, but one caregiver (CS: 08) opted for a drug shop over public health facility in the subsequent episode. One case (CS: 06) continued to take no action since the household “had no money to buy medicines”.

### Factors affecting decisions

Decision factors examined how caregivers’ previous experiences and advice from family members influenced their decision about drug used and sources accessed. All cases shared some examples of how their previous experiences had influenced their preference for certain drugs and/or treatment sources.

#### Past experiences influencing drug use

Previous experience(s) with a western medicine was an important factor influencing caregivers’ decisions for the current febrile episode, as were experiences with current episodes at influencing future decisions. We found cases with positive outcomes (CS: 06, 07, 08) were more likely to share both good and bad past experiences with drug use influencing current decisions, whereas those who had experienced a negative outcome (CS: 02, 03, 04) shared only good experiences with drug use. The following quotes illustrate why caregivers favored specific medicines.

*Okay I get him those medicines [Panadol® and Septrin®] because … they are the ones that I had [in the home] because I normally use and they work. (CS: 04)*

*I normally buy Panadol®, it's the one that I usually give them and they swallow…. At times I give them Panadol® and they get cured well. (CS: 06)*

*The fever [malaria] can match with quinine [uses quinine to treat]. (CS: 07)*

*I knew it because whenever I go with my child to the health unit, I am first given Panadol® and Coartem®. So, if I would pack some [Panadol® and Coartem®] … I can use them in case of a malaria attack…. From personal experience, whenever I use Panadol®, it relieves me of the fever and high temperature. (CS: 08)*

Similarly, caregivers avoided the use of certain medicines because of previous undesirable experiences with certain medicines. The following quotes are examples of some of these experiences:

*I used to see, if the child was just deteriorating … I would give [Coartem®]. Then I saw … it [Coartem®] … did not cure, did not cure at all, then I abandoned it [Coartem®] … without seeing any change…. (CS: 06)*

*We used to use chloroquine long time ago but whenever I would use it, it would itch my body, my whole body. So I no longer use that chloroquine. (CS: 08)*

#### Past experiences influencing source of care

Caregivers’ previous experiences with public health facilities and private outlets also contributed to their decision about which external source to use. With respect to negative experiences at public health facilities, caregivers discussed examples such as stock-outs or treatment failure influencing their decision to not return to that facility. Such poor experiences were not exclusive to cases with positive or negative outcomes (CS: 01, 03, 04, 06, and 07). As illustrated in Additional file 1: Table S1 and Additional file 2: Table S2, caregivers still visited public facilities despite their frustrations with services at public facilities (CS: 01, 04).

*[Speaking of an earlier fever episode with another child] On that particular day medicines had got finished from public health facilities [Namulo HC II and Kangalaba HC III], so that is when I took the child to the drug shop. But even when she treated, the child did not cure. So, I wait till they brought medicines to Nalusaga [Kangalaba HC III]. That is why I took [this] child to Nalusaga [Kangalaba HC III] for treatment since he [other child] had failed to cure with the treatment given to him by the drug shop. At Nalusaga [Kangalaba HC III], that is where the child got cured.… [When] I took my child to … Namulo HC II … he failed to improve so I stopped going there. (CS: 01)*

*The reason why I decided to go to the private [drug shop] is because I lost one of my children from that Busolwe hospital. (CS: 03)*

*They [drug vendors] provide for us with medicine, because at times we go to the government health units [and] medicines are not there, that's why we go to the clinics [drug shops]. (CS: 04)*

*In Naweyo [HC] when you go, when you go at times, you find that there is no health provider, and at times when you find the health providers, he writes for you [prescribes] and at times tells you that there is no medicines then you come back empty-handed or with nothing. (CS: 07)*

#### Advice influencing treatment sources

Advice from family or friends also influenced caregivers’ decision of where to seek external treatment. All caregivers cited receiving such advice either during the initial phase of the illness or during subsequent actions when the child had not improved. Most caregivers, three of the five with negative outcomes (CS: 02, 03, and 05) and all with positive cases (CS: 06, 07, 08), mentioned taking the advice given to them. Comparing the quotations with caregivers’ actions in Additional file 1: Table S1 confirms that such advice was followed.

*… [in] 2010 … we were told [by friends] about tablets that treat convulsions [from fever], that those tablets were found in Busolwe [hospital]. So, that is when we went to Busolwe to obtain those tablets. (CS: 02)*

*My mother told me not to dress up the child in any clothes [because of fever] but to wrap the child in a baby shawl and proceed directly to the health unit [drug shop]. When he convulsed, I ran very fast and [wrapped] wet cloth over his body. I then looked for a bicycle and rushed him to a [drug shop] in Busolwe. (CS: 03)*

*My husband used to tell me that we take the child to the clinic [HC]. Now, when the illness attacks the child and it was severe … [I] discuss with my husband and … go to the clinic [HC]. (CS: 05)*

*Now, the person [caregivers’ sister] tells me that take the child to the health unit [drug shop] and the child will cure of fever [malaria]. (CS: 06)*

*So when … I knew that it was fever and I called the child’s mother [the wife], the mother also touched, touched her and saw that she was badly off and I told her we take the child to the health unit [drug shop] maybe the health provider can help us. (CS: 07)*

*He [the husband] tells me that take the child to health unit [HC]. (CS: 08)*

#### Advice given to caregivers influencing treatment choices

Advice from family and friends also influenced caregivers’ initial treatment choices for their child. Three of the cases, representing both positive and negative outcomes (CS: 02, 03, 08), who were given advice about which source to access also spoke about receiving advice on how to treat their child. The only three cases to use traditional remedies (CS: 02, 03, 08) stated that they had done so because on advice received by a friend, mother, or grandmother. Only one caregiver (CS: 02) reported using a western medicine (quinine) at the advice of people she met on the road and at boreholes. Others used western medicines which had previously been recommended to them by health providers.

*There is a friend of mine who had a child who also used to convulse [from fever], so that friend of mine is the one that advised me to use the onion [so] I pounded the onions, had her drink some and smeared the rest on her body…. I would narrate [tell] the child's illness to her [my friend] and she would tell you what to do. (CS: 02)*

*Others [people from the village who she met on the road and at boreholes] would tell me that “I also had a child that used to convulse like yours, I continued to give her that quinine syrup until she got fine/cured”…. So, we used to give her the quinine.… We continued buying and giving her that quinine syrup. They [people from the village] gave us advice to continue giving her that quinine syrup so whenever the syrup would get finished, we would still buy more quinine syrup and give it to her. (CS: 02)*

*I got a wet cloth and pass it over his body again and again. [Then] my mother and mother-in-law said “if you pound onion and smear it all over his body, because it could be evil spirits [causing the convulsions] and the evil spirits can smell the onions and they run off. [So] I got onions and smear them all over his body. (CS: 03)*

*I was at my parents' home when the child fell ill. I had gone to mourn a dead relative when the child was ill. So at the funeral, my grandmother advised me to use mululusa. (CS: 08)*

Caregivers also relied on advice given by health providers during previous interactions. Two of the cases with positive results (CS: 07, 08) and one whose child had a negative outcome (CS: 04) initially managed their child using home management with a western medicine based on advice received from health professions during a previous interactions. Although only one caregiver (CS: 08) indicated she had been told been told by a health provider to use Coartem® when she herself got sick from malaria, so she believed it to be appropriate for her child. The advice for cases 04 and 07 did not follow the government recommendations for treating malaria (Additional file 1: Table S1).

*In the health unit, the health providers teach us to use this [Panadol®] and this [Septrin®]. (CS: 04)*

*The health provider [Drug Shop Vendor] told me, that this one [Panadol®] reduces painful body. (CS: 07)*

*I learnt [about Coartem®] because I also normally fall sick. So, when I fall sick and go to a health provider and explain to her about my illness, she tells me to go and swallow Coartem®, Panadol® and the other tablets for coughing whose name I do not remember. She tells you that “for every tablet … you will notice the body heat and fever being relieved”. So on that first day when I noticed [child had fever] I gave him the Coartem®. (CS: 08)*

### Challenges while seeking care at public health facilities

Treatment-seeking at public health facilities was associated with several challenges, with all caregivers mentioning one or more of these challenges. Challenges made up the largest part of the case study discussions and were not always specific to the current febrile episode in question. Despite the many challenges expressed by caregivers, a large majority took their child to public facilities on multiple occasions when their child did not improve. The most frequently voiced challenges included getting to a public health facility and the quality of service once at the public facility. Examples of challenges encountered once at public facilities included regular medication stock-outs, getting the runaround by the health staff, incivility of the staff, and the cost of medicines (Additional file 2: Table S2).

#### Access to public health facilities

Lack of access to public health facilities resulting from long geographic distances was a major concern for all caregivers as most often had to walk on foot to get there. Caregivers’ frustration with access is illustrated in the following three quotes:

*The challenge I face was to be referred to Businghu [Busiu Hospital in Mbale] yet [because] I did not have the means to get there. So I kept thinking about what to do. So then, I asked my husband about what we could do…. Transportation from Nalusaga [Kangalaba HC III] to Businghu costs 5,000 UGX using a taxi…. So it was my husband that found a way out. I guess he borrowed money from a friend. So with that money we went to Businghu. (CS: 01)*

*We walk [7 miles to Busolwe], we walk with our feet, that is when we have failed to get money. At times, we ride on the bicycle but when we fail to use the bicycle, we walk on foot to the hospital to get the medicines. (CS: 02)*

*It is there the clinic [drug shop] that was near, the other one [the HC] is far. And also I was challenged in form of money to go to the health unit. (CS: 07)*

#### Availability of health providers

Further complicating access to treatment was the unavailability of health providers when caregivers arrived at public facilities. Challenges of low staffing or absenteeism were experienced by several of the caregivers (CS: 02, 03, 04, 05, 07, 08).

*The challenge I faced is that I got there before the health providers. The health providers were not at the health unit by the time I got there. It was open and there was an askari [watchman] but the health providers had not yet come. That is the challenge that I faced. But as soon as they [the health providers] got there [30 minutes later], they worked on my case. (CS: 02)*

*Our health unit has only one health provider, I went there around 10:00 am and by 2:00 pm my child had not received any medical attention…. [Eventually] She touched and felt the boy's body heat with the back of her hand so with that, I guess she recognized what she should do with the child and the medicines to give for treatment. (CS: 08)*

#### Access to medicine

Once at a public health facility, a primary complaint voiced by six of the caregivers was unavailability of medications (commonly referred to as “stock-outs”) at the Level II and level III public facilities (CS: 01, 02, 04, 05, 06, 07). Additional file 2: Table S2 shows that blood tests were rarely taken, but when performed at all, were mostly available at higher level public health facilities, thus limiting the spectrum of care available at lower level public facilities.

*There are those [health facilities] that are nearby but I don’t go … because at times the medicines are finished … so you say let me obtain help from the other HC. [Health providers at Nalusaga: Kangalaba HC III] told me that for us here, we do not have medicines for your child but you go to Businghu [Busiu hospital in neighbouring Mbale District], so then she wrote for me a reference letter and I went to Businghu. (CS: 01)*

*I found challenges in mostly when I went and found no medicines there. I used to take her to the clinic [HC] when the illness is severe…. (CS: 05)*

*… at times when you find the health providers, he writes for you [prescribes] and at times tells you that there is no medicines then you come back empty-handed with nothing. (CS: 07)*

#### Getting the runaround

Parallel to the issue of stock-outs is the “runaround” given to caregivers when a public health facility is out of medicines. In circumstances when public facilities are experiencing stock-outs, caregivers are either referred to another public facility to manage the child or they are sent to a private drug vendor with a prescription to purchase their medicines, resulting in further delay in treatment. Caregivers whose child experienced a negative outcome were those who most often described getting the runaround. (CS: 01, 02, 03, 04, 05). Case 01 provides a good instance of a caregiver being sent to multiple locations, first because of stock-out at a nearby facility, then because of suffering an irreversible adverse outcome from over-medication. In the end the caregiver is told there was nothing that could be done for her child.

*It started with fever and then the child started to convulse, so I took him to health unit at Nalusaaga [Kangalaba HC III]…. They referred me to Busiu hospital [in neighbouring Mbale District], so I took him to Busiu hospital … and they told me to go buy water for the drip. After they discharged us, the child showed improvement but then the child’s sight became poor, child could no longer see property so they told us that "maybe the quinine that was given to the child was too much"…. So then we took him to Tororo thinking that maybe the eyes had a problem, that maybe the eyes were sick. We went to Tororo to check/examine the eyes and the eyes did not have any infection and they told us that maybe the medicine was too much, the quinine that they injected the child with was too much…. [As] the child was curing … this hand [holds the boys left hand that is lame] became lame. And even now, something attacks the brain and the brain stops working well … and the child falls down.... So, then, that is how he is..... (CS: 01)*

*I went to Budumba HC III people told me that "there are health providers who occasionally go there. They treat children that convulse. They have medicines that they give out freely". So with that, I went there. I went there with my daughter who is ill and I spoke to the health provider who told me that those have providers that treat convulsing children no longer went to Budumba HC III so he told me to go to Busolwe. He told me to go to Busolwe hospital so that is when I started to go to Busolwe. (CS: 02)*

*At Nabiganda [HC II], we did not get any treatment. When we reached, they [the health providers] told us that "we are not going to treat the child". Take him to Mbale [hospital]…. They said that … the blood is finished [child is anemic] so you go to Mbale. When I reached the health unit [Mbale hospital] they told us to buy a … set and a cannula … [so can draw] the child’s blood … they removed blood from him and they told us we take it and test it [get blood cross-matched at laboratory] so that they get [right] blood [and] they can give him. (CS: 04)*

#### Negative experiences at public health facilities

All caregivers expressed some level of discontent with the level of services they received, as well as with how they were treated by health providers at public health facilities. Once again, these complaints were more pronounced among caregivers whose child experienced a negative outcome compared to those whose child had a positive outcome. The complaints commonly involved failure to receive appropriate services and lack of attention at health facilities.

*When they tested [the child’s blood], they did not tell me the illness that my child was suffering from. (CS: 01)*

*That unit at Bingo HC II, when I go there, I am given only Panadol®, they do not examine the child, they do not even measure the child's temperature…. They [health professionals at Bingo HC II] do not have enough capacity to handle such a condition of a child so that is why they refer you to Busolwe main hospital…. Since we smeared him with onions, we could not dress him up, but still the truth is that he had no clothes of his own…. The health provider said that "why have you brought the child when he is naked” [the health provider at the government facility was angry that the child was not clothed]. (CS: 03)*

*On reaching [Mbale, since] 11 o'clock they had not given us [the child] blood, the child at 1 PM he died. (CS: 04)*

*The health provider just looks at you and he feels proud … [like] he own things, goes and comes back. Even if you go, there is nothing you get, the health providers are proud.… So there is nothing you get, the way you have gone, is the way you come back [child is ignored and not treated]. (CS: 06)*

*The health provider at times is busy with his work, can take long or he is having other patients. So there, we face also a challenge there. (CS: 07)*

### Burden of cost

The cost burden associated with treating malaria in their young children was a significant concern for all caregivers in this study. Caregivers reported incurring a variety of costs during the process of seeking treatment, including costs associated with: having to purchase medications from private outlets during periods of stock-outs, taking transportation to reach a public facility, managing a child febrile episode when there is treatment failure or treatment (or lack of) results in negative outcomes.

#### The cost of medicines

Medication stock-outs at public facilities inevitably required caregivers to purchase medications from private vendors for the health provider at the facility to administer or the caregiver to give to their child at home. This resulted in significant medication costs to households, thus negating the existing national treatment policy requiring children five and under seen at public facilities be treated for free. All caregivers in this study mentioned experiencing such medication costs.

*Because there was no quinine syrup at Busaba HC III, they told me to go and buy from anywhere. So I went to that drug shop and I asked if they had the syrup and they told me that they had it and that it costs 2500UGX. (CS: 02)*

*I face challenges when I could go to Jabusiba [Nakwasi HC III] and there is no treatment and they could tell me to go and buy. [But] malaria could not improve and it came back, she [the child] started swelling … I had to take her back again to the HC. (CS: 05)*

*There are times when you go to the health unit and do not find any tablets there. So, if there are no medicines at the health unit and are lucky enough you find the health provider there, she can ask for a book [write a prescription] in which she write the tablets which you have to buy for your child's illness. (CS: 08)*

#### Costs associated with finding time and taking transportation

Making time and finding transportation to reach public health facilities or private clinics was another factor adding to the burden of cost. All caregivers mentioned the cost of transportation as a burden, except for one case with a positive outcome (CS: 08) who was given Coartem® from a public health facility to keep at home for future use. Getting the runaround and being sent to several public health facilities and drug shops in order to receive treatment adds another layer of transportation costs. In some cases, such costs deterred people from seeking care at public facilities.

*The challenges that I face were about money because transportation to Nalusaga [Kangalaba HC III] on a motorcycle costs 1500UGX …. The [next] challenge I face was to be referred to Businghu [Busiu hospital in Mbale] yet [because] I did not have the means to get there. Transportation from Nalusaga to Businghu, costs 5000UGX using a taxi…. I asked my husband about what we could do … he borrowed money from a friend. So with that money we went to Businghu. (CS: 01)*

*I had also footed, walked on foot up to the health unit .… With the transportation problem, if I have money, the motorcycle is easy to use but the problem is money. You may want to use the bodaboda [motorcycle for hire] but when you do not have the money. Don't you see?.... The bodaboda guys [motorcycles] tell you to pay 4000UGX to and from Busolwe hospital. (CS: 02)*

*The child had malaria when he was still very young, just one week old. We were reluctant to take him to the health unit … [because] we failed to get money for treatment.… So that is how this cerebral malaria started. (CS: 03)*

*Transport [is a challenge] in the way that you may not be having a bicycle. And so the children fall sick, you need to find a way to the HC. (CS: 04)*

*I lacked transport to reach me to other HC … in terms of money for getting a car, motorcycle, like that. (CS: 05)*

*I have never gone to Busolwe hospital … because it is far, and also for transport the money is too much [for transportation to Busolwe]. (CS: 07)*

#### Cost of managing negative outcomes

Another important contributor to the cost burden were costs associated with managing negative outcomes of a malaria episode. Children who survived with a disability presented further costs to their families by requiring medicines to treat impairments resulting from the severity of the illness or because of inappropriate management of the malaria episode. Three of these children (CS: 01, 02, and 03) survived but with a negative outcome, all required on-going treatment and time.

*So, when the child was curing in the process, this hand [holds the boys left hand that is lame] became lame. And even now, something … like epilepsy … attacks the brain and the brain stops working well … and the child falls down…. So, then, that is how he is, I look after him in such state. (CS: 01)*

*Those [convulsion] tablets are found in Busolwe hospital, they are distributed every Friday, they give you tablets to last a whole week. So every Friday, we get those tablets for her…. We walk [7 miles to Busolwe], we walk with our feet, that is when we have failed to get money. At times, we ride on the bicycle but when we fail to use the bicycle, we walk on foot to the hospital to get the medicines. (CS: 02)*

*The child's illness has caused us a lot of poverty because when it [convulsion] catches him, we do not have money. We have to work in other people's gardens to pay for the cost [medicines and transportation]. So now, I do not know what to do to make the child's illness to get cured for good. (CS: 03)*

#### Sources of money

A further primary hurdle faced by most caregivers who obtained treatment for their child was the difficulty in obtaining money. Caregivers stated they had to seek credit from private vendors, borrow money from friends, obtain money from their spouse and family, deposit household goods, do extra work in exchange for money or sell household goods to pay for treatment.

*[Sometimes] I deposited the gomesi [dress] as security and obtained money … [from] a woman at a bar in the trading centre … when I wanted to buy the medicine for injection and tablets…. [Another time] I borrowed a bicycle which my husband deposited [as] security…and then he used the money to pay for the tablets and other medicines and then he also paid for the bodaboda. After that is when I go to the health unit or the other private clinic at Busolwe [to buy the medicine]. (CS: 03)*

*I take her to a clinic in Lelesi [drug shop] and they treat her with quinine … and tell me to come back with the money later. Then afterwards I look for money. You go, then someone gives you a garden, you dig, then in exchange she gives you money [and you pay the shop]. (CS: 05)*

*I am challenged in a way of money…. If am to go to the health provider [public health facility], they will tell me that they need money [before give treatment] yet I do not have [so] I [have to] go to borrow and to help me treat the child. (CS: 06)*

### Burden of living with a negative outcome

Living with a negative outcomes resulting in irreversible physical and/or mental disabilities generates unique burdens for both the child and the household. Unlike the burden of cost which can be discharged in a season or so, physical and mental defects last for the lifetime of both child and household.

#### Effect on the child and the household

Negative physical effects on the child were the most common burden mentioned and included decreased mobility, sight, and mental capacity. Cases 01, 02, 03, and 05 all had children who experienced prolonged episodes of malaria. As illustrated in these caregiver reports, these resulted in considerable suffering for the child, and impacted caregivers by adding to the burden of cost, requiring constant and vigilant care, dealing with socially inappropriate behaviors from the child, and living with enduring sadness.

*So when the child was curing in the process, this hand [refers to boy’s left hand] became lame. And even now, something … like epilepsy … attacks the brain and the brain stops working well it throws him down and the child falls down. I guess you can see the scars due to that [child to several wounds and sores on the foreheads and at the chin]. So then that is how he is, I look after him and such state. (CS: 01)*

*Convulsions fever caused her to stop talking and the brain became affected, she became mentally disturbed, affected and ill. She used to speak everything, even sing. If she could still speak, she would have by now already welcomed you, greeted you. She used to even thank me for the cooking after she had a meal [caregiver is now very emotional, close to tears]. But from the time she stopped talking, that is the same time during which her mental capacity was lost, and now she is mentally unstable…. Before getting mentally incapacitated, she would go and defecate in the latrine, but right now she just defecates anywhere that she finds. It can even be here right now, she can come and defecates here…. She can even go and defecate in the house. (CS: 02)*

*Whenever he catches the malaria, I do not want to leave him all by himself, I need to move with him all around [wherever] I go…. I go with him. When he gets a severe attack, and yet I have got no money, [I] still and move with him wherever I go [even when gardening]…. He [still] suffers the convulsions. Stomach swells very much. Whenever he convulses, [stomach] too swells up and it can continue to swell up to three months nonstop. He also does not eat … as for the stomach that one failed to get back to normal. In fact, it only continues to swell and swell even more. [The child's stomach is very swollen like a balloon]. That child's illness has caused us a lot of poverty because when it catches him, we do not have money. (CS: 03)*

*Body swells, both her [the child’s] legs and her hands. The cheeks would also swell, she would have diarrhea, she would cough, mucus would come out of the nose and her body would get very hot…. She continuously fell sick. God himself decided to take her. But I tried a lot to give her treatment and I was defeated, then the child died. (CS: 05)*

#### Knowledge of government recommended medicine

Caregivers reported a variety of beliefs about using western medicines or traditional remedies to treat malaria in young children. Preference for western medicine over traditional medicines to treat malaria was expressed by all caregivers, although some did mention traditional remedies also had role in some instances. Seven of the cases (CS: 01, 03, 04, 05, 06, 07, 08) acknowledged hearing about ACTs (or Coartem®), but only one (CS: 04) of the four cases asked to name the government recommended antimalarial correctly stated ACT/Coartem® (Table 3). Thus, not all caregivers who had heard of ACT or Coartem® recognized that Coartem® was an example of ACT, and not everyone was familiar with ACT or Coartem® being the government recommended antimalarial (Table 3). Additionally, only two sought out Coartem® to keep at home for future use (CS: 01, 08) which they initiated as part of home management, and only one caregiver (CS: 08) requested that their child’s current episode be treated with an ACT (or Coartem®) when visiting an external source.

**Table 3:** Caregivers’ knowledge of government recommended medicine^a^

Knowledge questions → Cases (CS) ↓	Does CG know the first-line gov’t antimalarial?	Has CG heard of ACTs?	Has CG heard of Coartem®?	Does CG know Coartem® is an ACT?	Where did CG hear about ACTs?	Was child given an ACT during this malaria episode?		Where does CG usually obtain ACTs?
						From home (Yes/No)	From 1^st^ external source (Yes/No)	
	Positive Outcome: Child Recovered							
CS 06	No	No	Yes	No	N/A	No	No (DS)	PHF for free (not this episode)
CS 07	No	Yes (But gives wrong examples^a^)	No	No	• Drug shops • Gov’t health providers • Radio	No	No (DS)	No evidence ACT was ever obtained
CS 08	N/A	N/A	Yes	N/A	N/A	Yes (leftover)	Yes (PHF)	PHF for free
Negative Outcome: Child Survived but with Deficits								
CS 01	N/A	Yes	Yes	No	• Gov’t health providers • Radio	Yes (leftover)	No (PHF)	PHF for free
CS 02	No	No	No	N/A	• Not heard	No	No (PHF)	Not for this index child
CS 03	N/A	Yes	No	No	• Radio	No	No (DS)	PHF for free (not this episode)
Negative Outcome: Child Died								
CS 04	Yes (Says ACTs & Coartem®)	Yes	Yes	Yes	• Gov’t health providers • Radio	No	No (PHF)	PHF for free (not this episode)
CS 05	N/A	N/A	Yes	N/A	N/A	No	Yes (PHF)	PHF for free

*Abbreviations*: Artemisinin Combination Therapy (*ACT*); Caregiver (*CG*); Government (*gov’t*); Drug Shop (*DS*); Not Applicable (*N/A*) - In some of the interviews these issues were not discussed; Public Health Facility (PHF)

^a^Examples given: Antibiotics; Aspirin®; quinine; chloroquine

*I know that the Western [medicines] work better than the traditional [because] I know that [Coartem®] works on malaria. (CS: 01)*

*[Western medicine] works speedily/fast and traditional treats but not much…. I know also that traditional ones also work … here in the village, we have our local or traditional medicine … if she vomits, they help her in not vomiting … it works [the traditional]. (CS: 07)*

*Myself. I knew that Coartem® also would play an important role in child's body…. I had got the Coartem® from the health provider at Muhuyu HC II because she is my husband’s friend. Previously, the child had been ill and had been given Coartem® to give to him. But on top of that, the health provider gave me other [additional] Coartem® to use later on in the future just in case any of my child got sick with malaria…. I learnt because I also normally fall sick … when I fall sick and go to a health provider and explain to her about those conditions, she tells me to go and swallow Coartem®. (CS: 08)*

Table 3 summarizes caregivers’ knowledge about ACT (and Coartem®), whether the child received it, and where they normally obtained it. We found that no febrile illness was treated in accordance with government malarial policy: receiving a confirmatory diagnosis for malaria and initiating a first-line antimalarial (quinine for those less than four-months and ACT for those greater than four-months) within 24 h of fever onset. While seven of these eight children were older than 4 months at the time of the febrile episode, and therefore eligible to receive an ACT, only three ever received it during the course of their illness.

### Lessons learned and suggestions for future improvements

Caregivers reported they had learned from this experience, and discussed how this will change their future treatment-seeking behaviors. They also made suggestions about what they believed need to occur within the health system to improve malarial treatment for children five and under.

#### How this experience changed caregivers’ treatment seeking behaviors

The experiences from this febrile episode influenced treatment-seeking behavior for all caregivers whose child had experienced a negative outcome (CS: 01, 02, 03, 04, 05), and all had several things to say during this portion of the interviews. Only one caregiver whose child had a positive outcome (CS: 08) participated in this discussion. Only one caregiver whose child survived with a disability (CS: 03) said she would not treat her children with quinine. One caregiver (CS: 01) stated that while she herself would not give quinine she would give it if it was recommended by a health provider, even though she now believes Coartem® is the best.

*I know that it [Coartem®] works best on malaria … I cannot use quinine again because I got him lame. Now I take him to a health provider who would know how to handle my son’s malaria…. [However] If it is a health provider [who recommends it], I do not refuse to use the quinine because it is the rule from the health provider. (CS: 01)*

*I have learnt that I should not use lots of quinine. I was told about the effects of quinine so now I also know that quinine damages the brain and mental capacity if you use it in large amounts. [Now] when she gets malaria, we give her these very [convulsion] tablets that we obtain from Busolwe hospital on Fridays. (CS: 02)*

*Nowadays when he gets sick did normally do not give him [quinine] injections…. I bathe him. If I have money, I get him some Panadol® so that the fever is relieved a bit. (CS: 03)*

Both caregivers whose child died said they would initiate treatment more promptly, one (CS: 05) by making sure she had Coartem® at home to initiate as home management, the other (CS: 04) still remained uninformed about the first-line antimalarial treatment.

*I [have learned to quickly] take her to the health unit. Okay I [get] him those tablets [Panadol® and Septrin®] because one time I had gone and they are the ones that I normally get for treatment. I even use these for myself for treatment. (CS: 04)*

*Okay when it comes [fever], I usually have Coartem® in the house, so when the sickness comes, it is the one that I usually give before I rush her to the health unit. (CS: 05)*

Two of the cases, one with a positive outcome (CS: 08) and one with a negative outcome (CS: 02), stated that they had learned from their experience to not use traditional remedies.

*I used [traditional meds] but they failed so I give up on them. (CS: 02)*

*I used it but it [mululusa] did not heal my child. So now, I do not use it. I realized that it [mululusa] wastes time. (CS: 08)*

Three of the five cases with a negative outcome (CS: 02, 03, 04) indicated they would in the future seek care from a public health facility.

*Now, what I have learnt is that if any other child has or suffers a convulsion, I do not follow anyone's advice of what to use to treat the child. I know that I have to go to the health provider whom I explained to him he advises me accordingly. (CS: 02)*

*I [would] take her [immediately] to the health unit [previously treated the child at home and only took the child to a health unit when symptoms got worse]. (CS: 04)*

In addition to treatment-seeking behavior, most caregivers (CS: 01, 02, 03, 07, 08) discussed the need to improve preventive measures and overall well-being of the child.

*To sleep in a clean place. You should sweep where they sleep even if they only sleep on a papyrus mat. (CS: 02)*

*Not giving them cold food in the morning since it is cold, it has parasites. So it is better that you prepare and give children warm food like porridge to eat. (CS: 03)*

*If child has a poor appetite, always try to give him variety. If child fails to eat something, try and give him something else to eat. Do not just ignore the child without giving him something to eat. Try your best to give him various foods to eat until child gets something that he desires to eat. In that way, I am helping to give care to child who has malaria. So that the child doesn't stay the whole day hungry and yet he is suffering from the sickness as well. (CS: 08)*

Four of the five cases with negative outcomes (CS: 01, 02, 03, 04) mentioned that nets were an important way to improve caregiving. While only two of these four cases (CS: 01, 04) reported actually using nets in their home, one (CS: 01) qualified the use of nets by saying that there was no way to avoid malaria.

*They [children five and under] sleep under the net…. You cannot avoid or prevent yourself from a mosquito. At times, you can be seated and it comes and bites you. So in case you have medicine it helps us. (CS: 01)*

#### Suggestions for improving health delivery at the systems level

Caregivers’ suggestions for improving health delivery were directed toward alleviating challenges they faced when seeking treatment from public health facilities. Two suggestions made by half of the cases with both positive and negative outcomes (CS: 01, 02, 05, 08) were to bring health services closer to their home and to have more personnel at public facilities.

*I think that the government should help us and build health units that are nearby so that even us who stay for we can start moving shorter distances to the health units. (CS: 01)*

*So, at least, various roles can be shared up [have more staff to do the different tasks]. One health provider can be dispensing the tablets and the other health provider can be injecting, rather than just one person giving out tablets and at the same time injecting and at the same time receiving the patients. You can imagine, of about 30 people or 25 people, how can one health provider attend to all those people at once. (CS: 08)*

Four other caregivers (CS: 03, 04, 05, 07) suggested they were interested in being given more medicine by the public health facilities. Both cases 05 and 07 said that the types of medicine they want are ACTs. While case 05 reported she had heard of Coartem® and her child had received Coartem® at the public health facility; case 07 was less familiar with Coartem® but had been told it was the first-line government recommended antimalarial by the research assistant conducting the household survey as part of the larger study. Two caregivers (CS: 05, 06) also indicated that public health facilities need to provide them with information on how to better care for their child.

*They [public health facilities] should immunize for malaria. (CS: 03)*

*They [public health facilities] should bring for us help by giving and providing us with tablets and medicines … specifically Coartem® … in village…. [Additionally] I would love to be told when the child fall sick and doesn't want to eat what do I do. (CS: 05)*

*We want government to bring for us mosquito nets, and also those [ACT] medicines they give us and be in the house as first aid so that we know what to do for the child very fast. (CS: 07)*

## Discussion

While case studies do not allow generalizability to a large population, they do provide greater insight into individual behaviors. These case studies offer valuable testimony of eight caregivers’ circumstances, experiences and challenges with treatment-seeking in Butaleja District in rural Uganda. In our study we examined the sequential steps taken by caregivers to manage their child’s febrile illness, their experiences with the various sources they visited and challenges they encountered during this process. Overall, caregivers had a pluralistic approach to treatment, with many taking multiple steps in an attempt to resolve their child’s febrile episode and to avoid adverse outcomes. Despite the variability in outcomes – illness improved, negative outcome with disability and negative outcome resulting in death - caregivers generally shared similar practices, experiences and challenges.

### Home management

Similar to other regions in Uganda and sub-Saharan African countries, most caregivers in our study initiated treatment with home management either as a primary approach to treat the febrile illness or as a temporary measure before seeking treatment from an external source [17, 31]. Home management commonly involved the use of supportive treatment, traditional herbs, and/or western medicines kept in the home for future use. Home medicines were mainly sourced from private drug shops or public health facilities, and some instances were leftover from previous treatments. Only one caregiver opted to do nothing other than observe the child as their initial act. While just under half of the caregivers reported that they did not intend to seek external care when they initiated home management, all caregivers whose child did not improve sought treatment from an external source within the same/next day. The strategy to start with home management allows caregivers to wait in hope the less expensive home medicines will suffice, postponing direct and indirect expenses associated with seeking external treatment [32]. This approach also enables those wishing to seek further treatment to secure money, liquid assets, or find alternate source of labor to pay for these expenses [17, 31, 33–36]. The practice to seek help from external sources only when home management is perceived to have failed or if the child is presenting with symptoms reflecting severe illness such as vomiting or diarrhea, has also been observed in several other regions of Uganda, sub-Saharan African countries, as well as south Asia [17, 33–37]. Thus, despite caregivers reporting a preference for public health facilities, direct and indirect costs frequently compel them to start with home management [35, 37]. As evident in our study, the delay in seeking external care often results in the child’s condition progressing from mild to severe, resulting in negative outcomes such as irreversible disability or death.

### Use of multiple treatment sources

Getting the runaround at public health facilities was a common occurrence in our study. A greater proportion of caregivers in our study chose public health facilities over private outlets (ration of 5:3) for their first external source, although three of the five were later referred to private drug shops to purchase medications for the child. Consequently, more caregivers obtained their treatments from private outlets than public facilities. Furthermore, half of the caregivers required multiple visits to the same or different sources because their child either did not improve, the child went on to suffer an adverse outcome, or public facilities where they were visiting were experiencing stock-outs of medicines and/or blood supply. In a few instances, caregivers were referred to higher level public health facilities for more invasive treatment without any treatment initiated at the primary external source. Thus, similar to what other researchers in sub-Sahara Africa have observed, caregivers in our study vacillated between multiple sources, travelling long distances and incurring numerous costs in an attempt to find a cure [32, 38, 39].

### Use of multiple treatments

The use of multiple treatments to manage children was common in our study. This practice of polypharmacy by caregivers likely occurs in hope that at least one approach or synergistic effects from the combination of treatments will help cure malaria in their child [17, 32]. “Multiple treatments” included the use of multiple western medicines together, and sometimes in combination with supportive care and traditional remedies. While polypharmacy was common with home management, this practice was also observed with public health facilities and private drug shops. Our results are in line with other studies that have found traditional and modern treatments, both effective and ineffective, to coexist [17, 32, 40]. A rural study conducted in Mali found caregivers often sought care from HCs when home remedies did not alleviate symptoms, but then they returned to traditional therapy if treatment from the HC failed. In our study, one caregiver reported reinstating a traditional remedy when the child failed to improve, supplementing the treatment prescribed by the health facility.

### Delays in treatment

While most caregivers responded to their child’s symptoms on the same day with home management, some versions of home management no doubt caused delay in seeking treatment from external sources to later in the day or the next day. For many children this wait was too long, with some children’s condition progressing from mild to severe. In some cases the wait may have been longer as caregiver waited for home remedies to take effect. Appearance of illness at night was another deterrent to seeking immediate treatment at an external source, since caregivers waited for the sun to rise. Once at a public facility, medication stock-outs caused still further delays by requiring caregivers to secure additional funds and to travel further distances to a private outlet where medicines could perhaps be purchased. Other barriers encountered at public facilities by caregivers in our study included long wait times and unavailability of staff. Prompt treatment with antimalarial is defined by the World Health Organization (WHO) and the Uganda National Malaria Control Strategy as administration of treatment within 24 h of onset of fever [7, 41]. Research exploring health system practices in Uganda and other sub-Saharan African countries has confirmed delay in treatment to be a common occurrence, usually associated with lack of sufficient and appropriate staffing, long wait times, and unavailability of medications [33–36, 42–44]. Thus, despite the fact that half our study’s caregivers reported seeking care from a public facility within 24 h, treatment may not have been initiated within those same 24 h.

### Prompt diagnosis and treatment with appropriate antimalarial

Only a small proportion of eligible children in our study received an ACT from an external source (25 %), despite revised guidelines prompting its use, recent initiatives to improve its access from private licensed facilities, and more ambitious national targets for treating malaria. The Uganda National Malaria Control Strategy recommends that all suspected cases of malaria be confirmed diagnostically, and those testing positive receive a first-line antimalarial within 24 h of fever onset [7]. The target set by the Uganda Malaria Control Strategic Plan was to have 85 % of children under five receive correct treatment according to the national treatment guidelines with 24 h of the onset of symptoms by year 2010 [7]. For uncomplicated malaria, the first-line antimalarial consists of oral quinine for children younger than four months and ACT for those older than four months. For severe malaria, treatment is initiated with injectable quinine followed by oral quinine or ACT depending on the child’s age. Additionally, in circumstances where a child needs to be referred to another health facility, both the WHO 2010 guideline and Uganda National Malaria Control Strategy recommend initiating treatment with rectal or intramuscular artesunate, artemether injectable or intravenous quinine *prior to referral* [7, 41]. For most children in our study, diagnosis was primarily presumptive with only two children receiving a confirmatory blood test for malaria, one at a public facility and another at a private outlet. In two of the three instances where ACT was given, it was initiated as part of home management. However, only one child was later maintained on the ACT when seen at the public health facility, the other was switched to oral quinine. Overall, one caregiver reported initiating ACT after visiting a public facility. Thus, in this study, public facilities were not dependable sources for receiving treatment as outlined by the national malaria policy. Children were no more likely to receive an appropriate antimalarial treatment if they were first seen at a public facility as opposed to a private outlet. The low use of ACTs in our study is in line with national and regional findings in Uganda which show the usage of ACT to be at 21 % in 2009 and 44 % in 2012, with an even lower proportion demonstrated to receive an ACT within the same/next day (18 % in 2009 and 36 % in 2012) [45, 46]. For those children in this study who died from a protracted case of fever, one died in spite of receiving an ACT. One possible explanation for the death may be the lack of proper clinical assessment and overall management by the public facility. Since many childhood illnesses share common symptomatology such as fever, fatigue, weakness, nausea and diarrhea, diagnostic testing for malaria is integral to a child receiving appropriate treatment. As observed in this study and reported by other, in circumstances where quality of services are poor and public health facilities are not adequately resourced, experiencing a negative outcome is not only inevitable but unfortunately predictable, suggesting the need to strengthen case management [39, 43].

### Stock-outs of ACT at public health facilities

For children in our study, it is possible that public health facilities may not have prescribed an ACT because they were already aware that few private drug shops -- which are mostly unlicensed in Butaleja District -- carry it. With funding from the Global Fund, Uganda implemented a national program in Spring of 2011 to disseminate ACTs through licensed private outlets at a subsidized cost [27]. However, purchase of ACTs from a licensed private outlet was beyond the reach of many caregivers, as most are poor and cannot afford to travel long distances to licensed private outlets. Half of the caregivers in our study relied on public health facilities to obtain an appropriate antimalarial, and the use of unlicensed private outlets was in large part due to unavailability of medicines at public facilities. In fact two caregivers in our study reported obtaining ACTs from public health facilities well in advance of an illness and keeping it at home for future use because of frequent stock-outs. Thus, in Butaleja District public facilities were not reliable sources of ACTs; and while unlicensed private vendors were an important source of antimalarial treatment, they were not licensed to sell ACTs.

### Caregivers prefer public health facilities despite challenges

Distances to health facilities were a note of concern by all caregivers, as many lacked resources such as money and transportation to travel to these facilities. Among those who were able to reach a health facility, they were further burdened by long wait times, medication and supply stock-outs, staff shortages, inattentive staff, staff that exhibited poor attitudes, and staff who delivered an overall poor quality of care. Despite such unpleasant experiences, many caregivers still expressed greater preference for public health facilities, with just over a half choosing public facilities as their first external source when their personal attempts to resolve the illness failed. As has been suggested by others, caregivers’ preference for public facilities may be associated with the belief that providers at these facilities are more qualified and experienced than those at private outlets [17, 34, 47]. Knowledge of such obstacles impeding access to prompt and appropriate treatment at public facilities in Uganda is not new and have been reported by many others [17, 44, 48, 49]. Our study however points to the fact that such challenges continue in spite implementation of national malaria control policies and training of public providers to improve case management. The literature suggests that interventions more far-reaching than health provider training are needed to improve care at public facilities [17, 48, 49]. These include implementing regular and adequate supervision, improving worker motivations and perceptions, improving customer service and communication between staff and caregivers, changing political and economic environment and incentives, mitigating misappropriation of drugs to ensure continuous stock of ACT, and removal of ineffective antimalarials from facilities [17, 48, 49]. Until such time as practices at public health facilities meet guidelines, promoting the use of public facilities will remain problematic. Public health researchers and program implementers need to consider multifaceted approaches involving all key stakeholders – including both caregivers and unlicensed drug vendors, to improve prompt and effective management of malaria in young children.

### Recommendations

A number of interventions have been implemented in Uganda to strengthen case management within the regulated public and private settings. However, considering the central role of caregivers and of the unlicensed private sector in the management of fever for young children in rural settings, future initiatives need to be multifaceted by including caregivers and all services accessed by caregivers [15, 17, 45, 48, 50, 51].

In Butaleja District where a large proportion of the population relies on unlicensed outlets, the current policy to limit dissemination of AMFm subsidized ACTs to only licensed private outlets has not resulted in acceptable malaria management practices [27]. One option to address the current gap between policy and practice is to extend the ACT subsidy program along with the necessary training to the unlicensed private sector [15, 51]. An additional strategy to engage the unlicensed sector may be through the integrated community case management (iCCM) program. There have been recent national initiatives to bring public services closer to the community through the introduction of iCCM, although at the time of this study this service had not yet been introduced in Butaleja District. This program is aimed at delivering prompt and free treatment to children under five for malaria, pneumonia, and diarrhea, through the use of volunteer CHWs who are recruited by the community but supported and supervised by the public health system [7, 52]. While short-term studies have demonstrated care delivered by CHWs to be effective under study conditions -- where close supervision and uninterrupted supply of ACT is assured, long-term effectiveness still needs to be demonstrated [53, 54]. Some of the challenges levelled against the original Home Based Management of Fever Program (HBMF) upon which the iCCM is modelled include frequent medication stock-outs and absenteeism of CHWs [55]. Given that iCCM is governed under the public health structure and CHW model continues to rely on volunteers, it also runs the risk of being plagued by similar problems [54, 56]. Current data from national and regional studies indicate that to-date there has been a less than optimal uptake of CHW services through the iCCM program [45, 48, 50]. Whether this low uptake is a consequence of earlier negative experiences with the HBMF program still needs to be investigated. What we do know from previous treatment seeking behavior studies is that poor quality of care and/or stock-outs can undermine the potential impact of providing free health care [33]. Given that most unlicensed vendors are first and foremost part of the communities they service, one possibility would be to consider a CHW model that includes them. Such a model may mitigate the concern of medicine stock-outs afflicting public outlets -- since private vendors can offer a constant supply of ACTs; and it may alleviate concerns with absenteeism because of their on-going presence.

Shaping caregivers’ expectations and decisions may be another important element to meet national case management objectives [18, 20, 48]. For years social scientists have advocated that acceptance of any healthcare intervention will ultimately depend on individuals’ accepting and demanding such interventions [57]. Accordingly, in circumstances where caregivers lack the appropriate knowledge or conviction, the literature indicates that they will often limit the demand for effective interventions even when it is available [20, 58]. While most caregivers in this study reported hearing of ACT, there was a low level of awareness that ACT was the first-line antimalarial. It is therefore not surprising that most caregivers did not request an ACT when obtaining treatment. Other studies in rural eastern Uganda have also found caregiver to commonly confuse non-antimalarials with antimalarials, with many opting for less effective therapies [17, 48]. Similarly, the low use of diagnostic tests to confirm suspected case of malaria in this study was also a concern. While we did not explore caregivers’ views of diagnostic tests, studies exploring community acceptance of routine diagnostic procedures to confirm malaria have identified disturbing findings. For instance, in Uganda, it was reported that community members generally feared getting their children’s blood tested for malaria because of concern that their child could get infected with HIV in the process, the blood would be used to test for HIV rather than malaria, or that the blood could end up in the wrong hands and be used for witchcraft [59]. Another study in Nigeria reported that while community members acknowledged the importance of testing for malaria, many remained doubtful about the reliability of the tests, especially when results are negative [60]. Given that those who are informed about the benefits of ACTs and of diagnostic testing are more likely to advocate for their use when seeking treatment for their child, this study clearly highlights the need to develop targeted education for caregivers around diagnostic testing and prompt treatment with first-line antimalarials [18, 59, 60].

### Limitations

Case study methodologies offer unique opportunities to explore and examine complex phenomena within natural contexts, and -- in the case of this study -- through multiple lenses [61]. The intent of this study was to document individual cases to capture thick descriptions of unique situations and to illustrate special attributes and aspects of care giving during a malaria episode, as well as to determine factors influencing caregiver practices as leverage points for defining a health promotion program. Despite their advantages, case study limitations need to be recognized and findings from this study need to be interpreted accordingly. First, case study methodologies generally use smaller sample sizes than found in most quantitative studies, thus this study makes no claim to generalizability. Second, case studies are subject to selection bias because each case is purposively selected by the research team to explore specific experiences. In our study, results represent insights from eight caregivers, thus the study findings are not necessarily representative of all caregiver experiences in Butaleja District. Third, the research was conducted retrospectively and therefore was subject to recall bias. While it was possible to minimize recall bias for those caregivers whose children had a positive outcome by limiting the discussion to the very last fever episode, it was difficult to minimize it from a methodological perspective for cases that experienced negative outcomes, as it required caregivers to reflect back to an episode that may have occurred over a year ago. Thus, recall bias may have resulted in omission or over-emphasis of certain discrete but relevant steps. Lastly, cases were recruited based on caregivers’ presumptive diagnosis of malaria. This could have resulted in sampling of some cases which may not have been malaria. However, given that fever commonly serves as a proxy for malaria in high endemic-regions where use of routine diagnostic procedures remains low, the sampling process followed real life practices.

## Conclusions

Findings from our study propose that these eight caregivers in Butaleja District generally shared similar practices, experiences and challenges when seeking treatment for children five and under, with few children ever receiving treatment in accordance with the Uganda national guidelines. To improve timely access to ACT, our results support the need to include all key stakeholders in future public health interventions aimed at improving malaria management in young children. Traditionally, training and certification has been limited to providers affiliated with the formal health system - public health facilities and licensed private outlets. However, given the weak infrastructure and limitations of the formal health system, focusing on these groups alone is not sufficient. As noted in this study and acknowledged by other researchers, unlicensed drug vendors as well as caregivers continue to play an essential role in the management of fever in young children in rural settings. Accordingly, future health delivery models need to consider how unlicensed vendors can be leveraged to help attain the national target -- to have 85 % of children under five receive antimalarial treatment as per guidelines. Additionally, future public health interventions need to improve caregivers’ capacity to take the necessary actions for their children by better informing households on how best to manage malaria in young children and to advocate for appropriate treatment.

## Additional files

Additional file 1: Table S1. Caregivers’ treatment seeking actions and sequence from point of initial symptoms^a^. (PDF 194 kb) Additional file 2: Table S2. Caregivers’ experiences with external sources^a^. (PDF 171 kb)

## References

[1] World Health Organization (WHO). World malaria report 2011. http://www.who.int/malaria/world_malaria_report_2011/9789241564403_eng.pdf. Accessed 1 Dec 2014.

[2] Liu L, Johnson HL, Cousens S, Perin J, Scott S, Lawn JE, Rudan I, Campbell H (2012). Global, regional, and national causes of child mortality: an updated systematic analysis for 2010 with time trends since 2000. Lancet.

[3] ter Kuile FO, Parise ME, Verhoeff FH, Udhayakumar V, Newman RD, van Eijk AM (2004). The burden of co-infection with human immunodeficiency virus type 1 and malaria in pregnant women in sub-Saharan Africa. Am J Trop Med Hyg..

[4] United Nations Children's Fund (UNICEF). Malaria and Children: progress in intervention coverage. http://www.unicef.org/health/files/Malaria_Oct6_for_web(1).pdf. Accessed 1 Dec 2014.

[5] World Health Organization (WHO). The global burden of disease: 2004 update. http://www.who.int/healthinfo/global_burden_disease/GBD_report_2004update_full.pdf. Accessed 1 Dec 2014.

[6] Steketee RW, Nahlen BL, Parise ME, Menendez C (2001). The burden of malaria in pregnancy in malaria-endemic areas. Am J Trop Med Hyg..

[7] Ministry of Health. Uganda malaria control strategic plan: 2005/06 - 2009/10. http://archiverbm.rollbackmalaria.org/countryaction/nsp/uganda.pdf. Accessed 1 Dec 2014.

[8] World Health Organization (WHO). The African summit on Roll Back Malaria. http://www.rollbackmalaria.org/microsites/wmd2011/abuja_declaration_final.html. Accessed 1 Dec 2014.

[9] United Nations. The Millennium Development Goals report 2008. http://www.un.org/millenniumgoals/2008highlevel/pdf/newsroom/mdg%20reports/MDG_Report_2008_ENGLISH.pdf. Accessed 1 Dec 2014.

[10] World Health Organization (WHO). World malaria report 2008. www.who.int/malaria/wmr2008/malaria2008.pdf. Accessed 1 Dec 2014.

[11] World Health Organization (WHO). Scaling up home-based management of malaria: from research to implementation. http://www.who.int/tdr/publications/documents/scaling-malaria.pdf. Accessed 1 Dec 2014.

[12] Greer G, Akinpelumi A, Madueke L, Plowman B, Fapohunda B, Tawfik Y, et al. Improving management of childhood malaria in Nigeria and Uganda by improving practices of patent medicine vendors. http://www.basics.org/documents/pdf/ImprovingMalariaMgmtPMVs.pdf. Accessed 1 Dec 2014.

[13] Hall S. People first: African solutions to the health worker crisis. http://www.chwcentral.org/sites/default/files/People%20First-%20African%20solutions%20to%20the%20health%20worker%20crisis.pdf. Accessed 1 Dec 2014.

[14] Zurovac D, Tibenderana JK, Nankabirwa J, Ssekitooleko J, Njogu JN, Rwakimari JB (2008). Malaria case-management under artemether-lumefantrine treatment policy in Uganda. Malar J.

[15] Rutebemberwa E, Pariyo G, Peterson S, Tomson G, Kallander K (2009). Utilization of public or private health care providers by febrile children after user fee removal in Uganda. Malar J.

[16] Ministry of Health Uganda, USAID, Makerere University School of Public Health, Health Systems 20/20. Uganda health system assessment 2011. http://health.go.ug/docs/hsa.pdf. Accessed 1 Dec 2014.

[17] Rutebemberwa E, Nsabagasani X, Pariyo G, Tomson G, Peterson S, Kallander K (2009). Use of drugs, perceived drug efficacy and preferred providers for febrile children: implications for home management of fever. Malar J.

[18] Goodman C, Brieger W, Unwin A, Mills A, Meek S, Greer G (2007). Medicine sellers and malaria treatment in sub-Saharan Africa: what do they do and how can their practice be improved?. Am J Trop Med Hyg..

[19] Goodman C, Kachur SP, Abdulla S, Bloland P, Mills A (2007). Drug shop regulation and malaria treatment in Tanzania--why do shops break the rules, and does it matter?. Health Policy Plan.

[20] World Health Organization (WHO). The Roll Back Malaria strategy for improving access to treatment through home management of malaria. http://whqlibdoc.who.int/hq/2005/WHO_HTM_MAL_2005.1101.pdf. Accessed 1 Dec 2014.

[21] Tawfik Y, Northrup R, Prysor-Jones S. Utilizing the potential of formal and informal private practitioners in child survival: situation analysis and summary of promising interventions. http://pdf.usaid.gov/pdf_docs/PNACP202.pdf. Accessed 1 Dec 2014.

[22] Wikipedia contributors. Butaleja District. http://en.wikipedia.org/wiki/Butaleja_District. Accessed 1 Dec 2014.

[23] Uganda Bureau of Statistics (UBOS). Higher local government statistical abstract: Butaleja District. http://www.ubos.org/onlinefiles/uploads/ubos/2009_HLG_%20Abstract_printed/Butaleja%20DLG%20Abstract-Final.pdf. Accessed 1 Dec 2014.

[24] Butaleja District Local Government NEMAN, United Nations Development Programme (UNDP)/Poverty and Environment Initiatives (PEI). District environmental policy. http://www.nemaug.org/district_policies/Butaleja_District_Environment_Policy.pdf. Accessed 1 Dec 2014.

[25] Uganda Bureau of Statistics (UBOS). 2010 Statistical abstract. http://www.ubos.org/onlinefiles/uploads/ubos/pdf%20documents/2010StatAbstract.pdf. Accessed 1 Dec 2014.

[26] Nanyunja M, Nabyonga-Orem J, Kato F, Kaggwa M, Katureebe C, Saweka J (2011). Malaria treatment policy change and implementation: the case of Uganda. Malar Res Treat.

[27] AMFm Independent Evaluation Team. Independent evaluation of phase 1 of the Affordable Medicines Facility - Malaria (AMFm), multi-country independent evaluation report: Final report. http://www.theglobalfund.org/documents/amfm/AMFm_2012IEPhase1FinalReportWithoutAppendices_Report_en/. Accessed 1 Dec 2014.

[28] Presentation to Global Health and Innovation Conference (GHIC). Access to effective antimalarial therapy remains low in rural Uganda - assessing unlicensed drug vendor contributions, presented on 12 April 2014 in Newhaven, Connecticut. http://www.uniteforsight.org/conference/speaker-schedule-2014.

[29] Strauss A, Corbin JN (1998). Basics of qualitative research: techniques and procedures for developing grounded theory.

[30] Golafshani N (2003). Understanding reliability and validity in qualitative research. Qual Rep..

[31] Williams HA, Jones CO (2004). A critical review of behavioral issues related to malaria control in sub-Saharan Africa: what contributions have social scientists made?. Soc Sci Med.

[32] Ellis AA, Traore S, Doumbia S, Dalglish SL, Winch PJ (2012). Treatment actions and treatment failure: case studies in the response to severe childhood febrile illness in Mali. BMC Public Health.

[33] Diaz T, George AS, Rao SR, Bangura PS, Baimba JB, McMahon SA (2013). Healthcare seeking for diarrhoea, malaria and pneumonia among children in four poor rural districts in Sierra Leone in the context of free health care: results of a cross-sectional survey. BMC Public Health.

[34] Uzochukwu BS, Onwujekwe EO, Onoka CA, Ughasoro MD (2008). Rural–urban differences in maternal responses to childhood fever in South East Nigeria. PLoS ONE.

[35] Malik EM, Hanafi K, Ali SH, Ahmed ES, Mohamed KA (2006). Treatment-seeking behaviour for malaria in children under five years of age: implication for home management in rural areas with high seasonal transmission in Sudan. Malar J.

[36] Kemble SK, Davis JC, Nalugwa T, Njama-Meya D, Hopkins H, Dorsey G (2006). Prevention and treatment strategies used for the community management of childhood fever in Kampala. Uganda. Am J Trop Med Hyg..

[37] Mohan P, Iyengar SD, Agarwal K, Martines JC, Sen K (2008). Care-seeking practices in rural Rajasthan: barriers and facilitating factors. J Perinatol.

[38] Lowassa A, Mazigo HD, Mahande AM, Mwang'onde BJ, Msangi S, Mahande MJ (2012). Social economic factors and malaria transmission in Lower Moshi. Northern Tanzania. Parasit Vectors..

[39] Deressa W (2007). Treatment-seeking behaviour for febrile illness in an area of seasonal malaria transmission in rural Ethiopia. Malar J..

[40] Hildenwall H, Rutebemberwa E, Nsabagasani X, Pariyo G, Tomson G, Peterson S (2007). Local illness concepts: implications for management of childhood pneumonia in Eastern Uganda. Acta Trop.

[41] World Health Organization (WHO). Guidelines for the treatment of malaria. http://www.ncbi.nlm.nih.gov/books/NBK254223/. Accessed 1 Dec 2014.

[42] Nuwaha F (2002). People's perception of malaria in Mbarara. Uganda. Trop Med Int Health..

[43] Kahabuka C, Kvale G, Hinderaker SG (2012). Factors associated with severe disease from malaria, pneumonia and diarrhea among children in rural Tanzania - a hospital-based cross-sectional study. BMC Infect Dis.

[44] Kiwanuka SN, Ekirapa EK, Peterson S, Okui O, Rahman MH, Peters D (2008). Access to and utilisation of health services for the poor in Uganda: a systematic review of available evidence. Trans R Soc Trop Med Hyg.

[45] ACTwatch Group. Household survey Uganda 2012 survey report. http://www.actwatch.info/sites/default/files/content/publications/attachments/ACTwatch%20HH%20Report%20Uganda%202012.pdf. Accessed 1 Dec 2014.

[46] ACTwatch Group. Household survey report (baseline) Republic of Uganda 03/09-04/09. http://www.actwatch.info/sites/default/files/content/publications/attachments/Uganda%20Household%20Baseline%2C%20ACTwatch%202009.pdf. Accessed 1 Dec 2014.

[47] Chuma J, Gilson L, Molyneux C (2007). Treatment-seeking behaviour, cost burdens and coping strategies among rural and urban households in Coastal Kenya: an equity analysis. Trop Med Int Health.

[48] Littrell M, Gatakaa H, Evance I, Poyer S, Njogu J, Solomon T (2011). Monitoring fever treatment behaviour and equitable access to effective medicines in the context of initiatives to improve ACT access: baseline results and implications for programming in six African countries. Malar J.

[49] Moszynski P (2010). Disappearance of drugs undermines Uganda's fight against malaria. BMJ..

[50] Rutebemberwa E, Kadobera D, Katureebe S, Kalyango JN, Mworozi E, Pariyo G (2012). Use of community health workers for management of malaria and pneumonia in urban and rural areas in Eastern Uganda. Am J Trop Med Hyg.

[51] Konde-Lule J, Gitta SN, Lindfors A, Okuonzi S, Onama VO, Forsberg BC (2010). Private and public health care in rural areas of Uganda. BMC Int Health Hum Rights.

[52] World Health Organization (WHO), United Nations Children's Fund (UNICEF). WHO/UNICEF Joint Statement: integrated community case management (iCCM). An equity-focused strategy to improve access to essential treatment services for children. http://www.who.int/maternal_child_adolescent/documents/statement_child_services_access_whounicef.pdf. Accessed 1 Dec 2014. 10.4269/ajtmh.2012.12-0221 PMC3748523 23136272

[53] Ajayi IO, Browne EN, Garshong B, Bateganya F, Yusuf B, Agyei-Baffour P (2008). Feasibility and acceptability of artemisinin-based combination therapy for the home management of malaria in four African sites. Malar J.

[54] Marsh DR, Hamer DH, Pagnoni F, Peterson S (2012). Introduction to a special supplement: evidence for the implementation, effects, and impact of the integrated community case management strategy to treat childhood infection. Am J Trop Med Hyg.

[55] Malimbo M, Mugisha E, Kato F, Karamagi C, Talisuna AO (2006). Caregivers' perceived treatment failure in home-based management of fever among Ugandan children aged less than five years. Malar J.

[56] Hamer DH, Marsh DR, Peterson S, Pagnoni F (2012). Integrated community case management: next steps in addressing the implementation research agenda. Am J Trop Med Hyg.

[57] Edberg M (2007). Essentials of health behavior: social and behavioral theory in public health (texts in the essential public).

[58] Goodman C, Kachur SP, Abdulla S, Mwageni E, Nyoni J, Schellenberg JA (2004). Retail supply of malaria-related drugs in rural Tanzania: risks and opportunities. Trop Med Int Health.

[59] Mukanga D, Tibenderana JK, Kiguli J, Pariyo GW, Waiswa P, Bajunirwe F (2010). Community acceptability of use of rapid diagnostic tests for malaria by community health workers in Uganda. Malar J.

[60] Ezeoke OP, Ezumah NN, Chandler CC, Mangham-Jefferies LJ, Onwujekwe OE, Wiseman V (2012). Exploring health providers' and community perceptions and experiences with malaria tests in South-East Nigeria: a critical step towards appropriate treatment. Malar J.

[61] Baxter P, Jack S (2008). Qualitative case study methodology: study design and implementation for novice researchers. Qual Rep..

