# Supplementary material for: Patterns of treatment-seeking behaviors among caregivers of febrile young children: a Ugandan multiple case study
Source: BMC Public Health. 2016 Feb 16;16:160. doi: 10.1186/s12889-016-2813-7 (PMC4755008; doi:10.1186/s12889-016-2813-7)
Supplement: Additional file 1: Table S1. — Caregivers’ treatment seeking actions and sequence from point of initial symptomsa. (PDF 194 kb) [file 12889_2016_2813_MOESM1_ESM.pdf]

Table S1. Caregivers' treatment seeking actions and sequence from point of initial symptoms[a]

	Positive Outcome: Child Recovered			Negative Outcome: Child Survived but with Deficits			Negative Outcome: Child Died	
**Cases →**	**CS 06**	**CS 07**	**CS 08**	**CS 01**	**CS 02**	**CS 03**	**CS 04**	**CS 05**
**1st Action**	**Time:** 12 am<br>**Setting:** Home<br>**Trigger:** "Difficulty breathing", "wheezing", "congested", weak", "not playing", "crying", "sneezing", "cough"<br>**Blood Test:** No<br>**Treatment:** None<br>**Outcome:** Breathing worse & thick mucus	**Time:** 9 am<br>**Setting:** Home<br>**Trigger:** "Shivering", "sweating", "heat", stomach "bubbling", "eyes were red", "vomiting"<br>**Blood Test:** No<br>**Treatment:** Panadol®<br>**Outcome:** Shivering ↓, no other improvement	**Time:** 7 am<br>**Setting:** Home<br>**Trigger:** "Not eating", "not playing", "crying", "hot body", "rash", "mucus", "cough"<br>**Blood Test:** No<br>**Treatment:** Wet cloth, mululusa, Coartem®, Panadol®<br>**Outcome:** Worse	**Time:** 10 pm<br>**Setting:** Home<br>**Trigger:** "Fever", "convulsing", "not breastfeeding/eating"<br>**Blood Test:** No<br>**Treatment:** Wet cloth, Coartem®, Panadol®<br>**Outcome:** Fever not relieved	**Time:** 4 am<br>**Setting:** Home<br>**Trigger:** "Fever", "eyes became red", "stayed there in such a state", "convulsing", " weak"<br>**Blood Test:** No<br>**Treatment:** Smeared pounded onions on body, quinine syrup<br>**Outcome:** Fever ↓, on-going convulsions	**Time:** 5 pm<br>**Setting:** Home<br>**Trigger:** "Vomit", "hot body", "eyes staring and convulsed", "stomach swollen"<br>**Blood Test:** No<br>**Treatment:** Smeared pounded onions<br>**Outcome:** Eyes back to normal but still looked like a dead person	**Time:** 4 pm<br>**Setting:** Home<br>**Trigger**: "Weak", "retching", "hot body", refused to eat, "breathless"<br>**Blood Test:** No<br>**Treatment:** Wet cloth, Panadol®, Septrin®<br>**Outcome:** Could not stand & had a weak neck	**Time:** 5pm<br>**Setting:** Home<br>**Trigger:** "Cough", "diarrhea", "vomit", "hot body"<br>**Blood Test:** No<br>**Treatment:** Wet cloth<br>**Outcome:** Fever ↓, then visited HC
**2nd Action**	**Time:** Next day (am)<br>**Setting:** Drug shop<br>**Trigger:** Previous symptoms + "fever"<br>**Blood test:** No<br>**Treatment:** Junior Aspirin® from drug shop x 3 days, no money to buy more medicine<br>**Outcome:** Fever resolved in 2 weeks	**Time:** Same day (evening) & next day (morning & evening)<br>**Setting:** Drug shop<br>**Trigger:** No improvement<br>**Blood Test:** Yes<br>**Treatment:** Quinine injection, quinine & Panadol® tablets<br>**Outcome:** Improved gradually	**Time:** Next day (10 am)<br>**Setting:** Muhuyu HC II<br>**Trigger:** Worsened<br>**Blood Test:** No<br>**Treatment:** Some kind of injection, Coartem®, Panadol®, Rene Coldease®<br>**Outcome:** No fever, cured in 3-4 days	**Time:** Next day (am)<br>**Setting:** Kangalaba HC III<br>**Trigger:** Previous symptoms + "fever"<br>**Blood Test:** No<br>**Treatment:** None, referred to Busiu hospital in Mbale for blood test. Told to feed milk & porridge<br>**Outcome:** Referred to Mbale hospital	**Time:** Same day (10 am)<br>**Setting:** Busaba HC III<br>**Trigger:** "Fever", "coughing", "convulsions"<br>**Blood Test:** Yes<br>**Treatment:** Septrin®, quinine tablets, Panadol® tablets, and told to buy quinine syrup<br>**Outcome:** Told has cerebral malaria (with convulsions), convulsions ↑ to 8-15 x day	**Time:** Next day (10 am) & following day (10 am)<br>**Setting:** Drug shop<br>**Trigger:** "Vomit", "convulsions", "hot body", "blank stare", "swollen stomach"<br>**Blood Test:** No<br>**Treatment:** Told has cerebral malaria. Injections x 2 days, tablets: ibuprofen, 4 green, 6 white, 8 Panadol®<br>**Outcome:** Cured in 2 weeks. Still has swollen stomach & convulsions	**Time:** Next day (7 am)<br>**Setting:** Nabiganda HC II<br>**Trigger:** Worsened<br>**Blood Test:** No<br>**Treatment:** None, told could not treat before receiving blood transfusion. Referred to Mbale hospital<br>**Outcome:** Travelled to Mbale	**Time:** Same day (6 pm)<br>**Setting:** Nakwasi HC III<br>**Trigger:** "Cough", "diarrhea", "vomit"<br>**Blood Test:** No<br>**Treatment:** Coartem®. Told to buy quinine syrup from drug shop<br>**Outcome:** Symptoms not improve, body started to swell & condition worsened
**3rd Action**	X	X	X	**Time:** Same day<br>**Setting:** Admitted in Mbale x 3 days<br>**Trigger:** Referred<br>**Blood Test:** Yes<br>**Treatment:** Blood transfusion. Told to buy water for drip, 6 quinine injections, paracetamol, quinine syrup x 2 weeks, plus other pills<br>**Outcome:** Improved but sight affected, left hand became lame, on-going convulsions	**Time:** 2 weeks later<br>**Setting:** Drug shop & Busaba HC III<br>**Trigger:** Daily convulsions 8-15 x day<br>**Blood Test:** No<br>**Treatment:** Quinine syrup daily x 1 year from drug shop & Busaba HC<br>**Outcome:** Child stopped talking after 1 year	X	**Time:** Same day (~2 ½ hours later at 11 am)<br>**Setting:** Mbale hospital<br>**Trigger:** Referred<br>**Blood Test:** Yes<br>**Treatment:** None started for 2 hours while at the hospital<br>**Outcome:** Waited for 2 hours for treatment - child died	**Time:** 2 weeks later<br>**Setting:** Nakwasi HC III<br>**Trigger:** Worse, body started to swell<br>**Blood Test:** No<br>**Treatment:** Coartem® plus quinine syrup from drug shop<br>**Outcome:** No improvement
**4th Action**	X	X	X	**Managing Negative Outcome**<br>**Time:** 2 days later<br>**Setting:** Tororo hospital<br>**Trigger:** Decreased vision<br>**Blood Test:** No<br>**Treatment:** None<br>**Outcome:** Told quinine caused child to become lame & affected eyesight	**Time:** 1 year later<br>**Setting:** Home<br>**Trigger:** Child not talking<br>**Blood Test:** No<br>**Treatment:** Stopped quinine, started mululusa & tomato leaves baths x 1 month, then gave up<br>**Outcome:** Month later visited Budumba HC III when a friend told her convulsion drugs available there	X	X	**Time:** 1 month later<br>**Setting:** Nakwasi HC III<br>**Trigger:** No improvement<br>**Blood Test:** No<br>**Treatment:** Coartem®. Told to buy quinine syrup & other medicines from drug shop<br>**Outcome:** Improved, but then fell ill again ~3 months later
**5th Action**	X	X	X	**Managing Negative Outcome**<br>**Time:** 1 month later<br>**Setting:** Mbale hospital<br>**Blood Test:** No<br>**Trigger:** Examine eyes/lame hand<br>**Treatment:** None<br>**Outcome:** Told nothing could be done: look after child, feed passion fruits & milk	**Time:** 5 months later<br>**Setting:** Busolwe hospital<br>**Trigger:** Referred from Budumba HC III<br>**Blood Test:** No<br>**Treatment:** Quinine injection, convulsion tablets refilled weekly<br>**Outcome:** On-going convulsions but less frequent	X	X	**Time:** ~3 months later<br>**Setting:** Drug shop<br>**Trigger:** Sick again & Nakwasi HC III closed<br>**Blood Test:** No<br>**Treatment:** For worms<br>**Outcome**: Thought was improving but later that day vomited blood & died

[a]Abbreviation Health Centre (HC).
