# Supplementary material for: Patterns of treatment-seeking behaviors among caregivers of febrile young children: a Ugandan multiple case study
Source: BMC Public Health. 2016 Feb 16;16:160. doi: 10.1186/s12889-016-2813-7 (PMC4755008; doi:10.1186/s12889-016-2813-7)
Supplement: Additional file 2: Table S2. — Caregivers’ experiences with external sourcesa. (PDF 171 kb) [file 12889_2016_2813_MOESM2_ESM.pdf]

Table S2. Caregivers' experiences with external sources[a]

	Positive Outcome: Child Recovered			Negative Outcome: Child Survived but with Deficits			Negative Outcome: Child Died	
**Cases →**	**CS 06**	**CS 07**	**CS 08**	**CS 01**	**CS 02**	**CS 03**	**CS 04**	**CS 05**
**1st Action**	**Nearest PHC Accessed:**<br>No<br>**Reason not using PHC:**<br>Stock-outs, told to go to drug shop, health professionals are rude, & cost of transport<br>**Used:** Drug shop ~ 1 mile	**Nearest PHC Accessed:**<br>No<br>**Reason not using PHC:**<br>Lack of staff, stock-outs, & cost of transport<br>**Used:** Drug shop ½ mile	**Nearest PHC Accessed:**<br>Yes<br>**Used:** Muhuyu HC II	**Nearest PHC Accessed:**<br>No<br>**Reason not using closest PHC:**<br>stock-outs<br>**Used:** Kalabangha HC III ~ 5 miles	**Nearest PHC Accessed:**<br>Yes<br>**Used:** Busaba HC III ~ 3 miles	**Nearest PHC Accessed:**<br>No<br>**Reason not using PHC:**<br>verbal abuse from health professionals, no injections given, lack of examination & stock-outs<br>**Used:** Drug Shop ~ 3 miles	**Nearest PHC Accessed:**<br>Yes<br>**Used:** Nabiganda HC II	**Nearest PHC Accessed:**<br>Yes<br>**Used:** Nakwasi HC III
	**Setting:** Drug shop<br>**Experience:**<br>• Took child ~ 12 hours from start of symptoms<br>• No blood test done<br>• No information about illness given<br>• Vendor suggested antimalarial syrup, but refused as had no money<br>• Caregiver requested Aspirin® for 3 days. No money to buy more drugs<br>• Cost was 100UGX, willing to pay up to 500UGX<br>• Continued to be sick but no additional medicines given. Gradually resolved	**Setting:** Drug shop<br>**Experience:**<br>• Took child ~ 9 hours from start of symptoms<br>• Blood test done but not told of results<br>• No information about illness given<br>• Bought medicines<br>• Cost was 2000UGX<br>• Improved gradually	**Setting:** Muhuyu HC II<br>**Experience:**<br>• Took child after 1 day from start of symptoms<br>• Had to wait 4 hours before being seen<br>• Was examined<br>• No blood test done<br>• No information about illness given<br>• Received free medicines<br>• Cured in a few days	**Setting:** Kalabangha HC III<br>**Experience:**<br>• Took child ~ 12 hours from start of symptoms<br>• Was examined<br>• No blood test done<br>• Told was anemic, had high fever<br>• No medicines given<br>• Referred to hospital in neighboring district of Mbale because had no blood to transfuse<br>• Subsequent action taken	**Setting:** Busaba HC III<br>**Experience:**<br>• Took child ~ 6 hours from start of symptoms<br>• Waited 30 minutes before health professionals arrived<br>• Blood test done<br>• Told had malaria & convulsions from fever<br>• Received free medicines & told to buy more from drug shop<br>• Not improved, convulsions worsened<br>• Subsequent action taken	**Setting:** Drug shop<br>**Experience:**<br>• Took child ~ 17 hours from start of symptoms<br>• Provider was busy but was provided immediate care because saw child was in a bad state, told others to wait while she treated the child<br>• No blood test done<br>• Examined, touched & looked at eyes<br>• Told to cover child with blanket , hold him so doesn't fall down, & to return the next day<br>• Bought medicines<br>• Cost was 13000UGX for day 1; 7500UGX for day 2<br>• Malaria resolved gradually, but stomach remained swollen & child continued to convulse	**Setting:** Nabiganda HC II<br>**Experience:**<br>• Took child ~ 15 hours from start of symptoms<br>• No blood test done<br>• Told was anemic<br>• Received no medicines<br>• Told would not treat because needed to first receive blood transfusion<br>• Child referred to hospital in neighboring district of Mbale<br>• Subsequent action taken	**Setting:** Nakwasi HC III<br>**Experience:**<br>• Took child ~ 1 hour from start of symptoms<br>• No blood test done<br>• No information about illness given<br>• Received free medicines & told to buy more from drug shop<br>• Did not improve<br>• Cost not shared<br>• Not improved, body swollen, could not eat<br>• Subsequent action taken
**2nd Action**	X	X	X	**Setting:** Busiu hospital in Mbale<br>**Experience:**<br>• Health professionals ignored her child even when asked for help<br>• Blood test done but not told of results<br>• Told to buy water for drip, quinine injections, quinine syrup for 2 weeks and other oral medicines, and then to come back<br>• Cost was 15000UGX<br>• Malaria resolved, but eye sight affected, could not see, left hand became lame, experiences convulsions regularly	**Setting:** Drug shop & visited Busaba HC III & Busolwe hospital x 1 year<br>**Experience:**<br>• Gave quinine syrup for 1 year on advice from neighbors, health professional, & drug vendor. But stopped when child stopped talking<br>• At Busaba HC III & Busolwe hospital given quinine injections, & bought quinine syrup from drug shop every 2 weeks<br>• Cost 2500UGX every 2 weeks<br>• At times sent away from Busaba HC III because of stock-outs<br>• At Busaba HC III & Busolwe hospital never referred for convulsions<br>• Blood test never repeated<br>• Malaria resolved, but left unable to speak and brain-damaged	X	**Setting:** Mbale Hospital<br>**Experience:**<br>• Took blood test but not told of results<br>• Caregiver asked to get blood cross-matched at the laboratory, then to buy blood & transfusion kit<br>• Child died before blood could be purchased<br>• Health professional gave no information about illness<br>• No treatment started during the 3 hours at the hospital, arrived 11 am & died at 1 pm<br>• After child died, body cleaned & wrapped, returned to family	**Setting:** Nakwasi HC III<br>**Experience:**<br>• Returned in 2 weeks, then frequently over 4 months. Last visit at drug shop because HC was closed<br>• Would improve some, but illness would come back<br>• Same treatment at HC as 1st action<br>• Free medicines & told to buy more from drug shop<br>• No blood test<br>• No information about illness given<br>• Last visit at drug shop, given tablets for worms, child died later that day

[a]Abbreviations: Public Health Centre (PHC); Health Centre (HC); Ugandan Shilling (UGX).
